# Analysis of differences in cigar tobacco leaves from different regions based on GC-IMS and LC-MS metabolomics techniques

**DOI:** 10.3389/fpls.2025.1557190

**Published:** 2025-04-29

**Authors:** Dan Chen, Liang Feng, Haowei Sun, Risheng Zhong, Chunqiong Wang, Xiaowei Zhang, Ke Zhang, Ling-duo Bu, Wanlong Yang, Kai Liu, Haitao Chen, Shuqi Wang

**Affiliations:** ^1^ Yunnan Tobacco Quality Inspection & Supervision Station, Kunming, Yunnan, China; ^2^ Beijing Key Laboratory of Flavor Chemistry, Beijing Technology and Business University, Beijing, China; ^3^ Yunnan Tobacco Company, Yuxi, Yunnan, China; ^4^ Yunnan Oriental Tobacco Company, Ltd., Kunming, Yunnan, China

**Keywords:** LC-MS, regional differences, chemical composition, volatile compounds, chemometrics

## Abstract

This study aimed to investigate the differences in volatile compound composition and metabolites in cigar tobacco leaves from different regions of Yunnan. Cigar tobacco leaves from various regions and varieties in Yunnan were analysed using gas chromatography-ion mobility spectrometry and non-targeted metabolomics techniques. Results showed that 109 volatile compounds, including 26 esters, 17 aldehydes, 14 alcohols, 14 ketones, 9 olefins, 5 pyrazines, 4 ethers, 4 acids and 16 others, were identified in cigar tobacco leaves. Through GC-IMS analysis of volatile compounds in cigar tobacco from 10 regions, 1-methylethyl acetate, diethyl acetal, butanal, 1-hexanol, pyridine, and toluene were identified as common compounds with consistently high content across all regions. For regional characteristics, BS-Y1-1 is featured by high levels of 2,3-diethyl-6-methylpyrazine and phenylacetaldehyde; PE-Y2 shows the highest content of 3-methyl-1-pentanol; and WS-Y38 is characterised by significantly high levels of butan-2-one. These differences reflect the uniqueness of volatile components in cigar tobacco from different producing areas. The volatile compounds in Yunnan cigar tobacco leaves were greatly influenced by the origin and species, with cigar tobacco leaves from the Baoshan region differing from those in other regions. According to the Kyoto Encyclopedia of Genes and Genomes enrichment analysis, amino acid metabolism, nucleotide metabolism and glyoxylate and dicarboxylate metabolism were the main metabolic pathways, and their metabolites contributed to the formation of flavour in Yunnan cigar tobacco leaves.

## 1 Introduction

Cigar is a tobacco product made from air-cured and fermented tobacco, and the flavour of cigar is different from that of cigarettes (Liu et al., 2021). The aroma of cigar tobacco is primarily influenced by the tobacco itself and the fermentation process (Zhao et al., 2007). The detection and analysis of the aroma components of cigar tobacco can directly reflect the quality of raw materials and the characteristics of the fermentation process, which can provide important theoretical support for the production and quality control (QC) of cigar tobacco (Hu et al., 2023). The metabolites produced by cigar tobacco after fermentation are affected by various factors, such as the environment, variety and cultivation method. Among these, environmental conditions greatly influence the tobacco leaves. Therefore, the metabolites of cigar tobacco fermentation may differ significantly across regions (Wu et al., 2013). Some scholars have unilaterally focussed on volatile components or non-volatile substances of cigar tobacco, while others have measured specific substances that contribute significantly to the flavour of cigar tobacco (Li et al., 2023, 2024b). Studies examining the metabolites of volatile and non-volatile components in cigar tobacco from different regions are relatively rare.

Leading countries in the production of cigar tobacco leaves include Cuba, the Dominican Republic and the United States, with specific varieties suited to the characteristics of each production area. China’s Yunnan region, which shares a similar latitude with Cuba, has favourable ecological conditions, including abundant sunlight, well-coordinated temperature and humidity and fertile soils that promote the growth of high-quality cigar tobacco. Cigar tobacco research in China started relatively late, with most varieties being foreign hybrids or locally cultivated varieties. However, Yunnan has independently selected and bred several high-quality varieties, such as ‘Yunxue No. 1’ and ‘Yunxue No. 2’ (Xiaohong et al., 2010). The tobacco from these varieties has the typical characteristics of cigar tobacco, including good elasticity, toughness, tensile strength, flavour, sweetness and combustibility, making it highly promising for future development. Hou et al. analysed the aroma of cigar tobacco from different origins using diversity evaluation methods and found that the green aroma of Yunnan cigar tobacco is prominent, with hints of sweetness and woodiness, while the core of the cigar is mainly sweet with green notes and a nutty aroma (Hou et al., 2024). However, compared to foreign high-quality cigar tobaccos, Yunnan cigar tobacco still faces challenges, such as lacking a typical cigar flavour profile, discomfort and poor combustibility.

Gas chromatography-ion mobility spectrometry (GC-IMS) has been widely used in the analysis of volatile flavours in food products (Wang et al., 2020; Yang et al., 2023). The method is based on an easy-to-operate headspace injection without complex sample pretreatment and combines the high separation capability of GC and the fast response performance of IMS. It offers advantages such as rapidity, non-destructiveness, accuracy and high throughput (Yin, 2021). GC-IMS has been used to identify volatile compounds in tobacco leaves (Liu et al., 2024a). Non-targeted metabolomics is a large-scale, systematic analysis of metabolites in samples based on high-throughput detection and multivariate data processing. It is characterised by unbiased and holistic detection, reflecting metabolite changes to the greatest extent possible (Yin, 2021). By detecting the changes of metabolites in organisms under different conditions (such as developmental stages, stress treatments or physiological states), and analysing metabolite content and pathways, it is possible to reflect the metabolism level, physiological state, and disease progression of organisms. This approach has been widely used in various fields, including plants (Shen et al., n.d), animals (Mukherjee et al., 2023) and microorganisms (Zhang et al., 2016). Li et al., (2024a). used metabolomics technology to analyse the metabolic differences and formation mechanisms in open-fire smoked tobacco, finding that the differential metabolites of open-fire smoked tobacco contained many acidic metabolites.(Wang et al., 2024). explored the effects of different light durations on the growth and quality of roasted tobacco at the metabolomics level. They found that light durations may regulate metabolic pathways, including phenylpropanoid biosynthesis, pyrimidine metabolism, amino acid-related pathways and the synthesis of nicotinic acid-derived alkaloids in roasted tobacco. These pathways may influence the growth, development and quality of roasted tobacco.

In this study, based on metabolomics research methods, both volatile and non-volatile components of cigar tobacco leaves from different regions were analysed using GC-IMS and high-performance liquid chromatography (HPLC). Multivariate statistical analysis was used to identify differential metabolites between different tobacco leaves. Finally, the volatile and non-volatile differential metabolites were used as the foundation for a comprehensive analysis and evaluation of the compositional differences among various cigar tobacco leaves. This research is significant for developing Yunnan cigar tobacco leaves and the promotion of raw materials for domestic cigars.

## 2 Materials and methods

### 2.1 Plant materials

**Figure 1:**
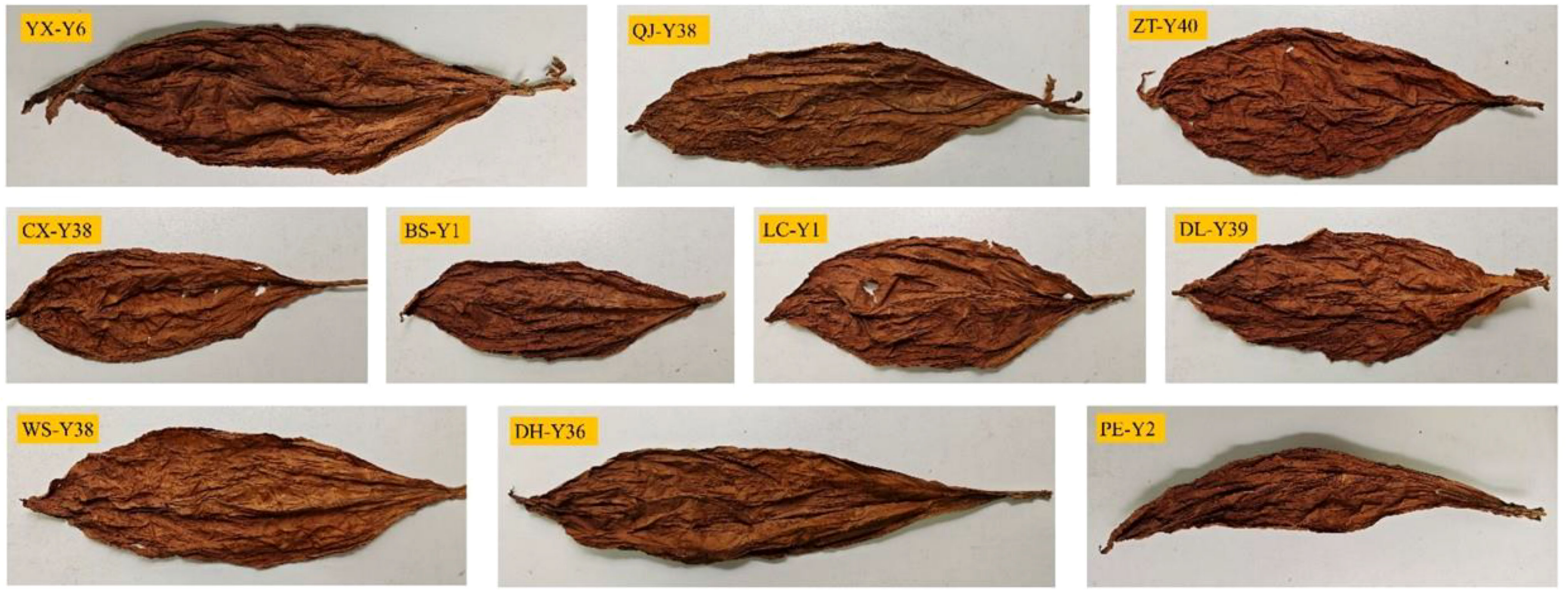
Appearance of tobacco from different origins.

The cigar tobacco samples were provided by Yunnan Tobacco Quality Supervision and Testing Station. Ten varieties of cigar core tobacco samples from 10 regions (Baoshan: BS-Y1-1, Cuxiong: CX-Y38, Dali: DL-Y39, Dehong: DH-Y36, Lincang: LC-Y1, Puer: PE-Y2, Qujing: QJ-Y38, Wenshan: WS-Y38, Yuxi: YX-Y6, Zhaotong: ZT-Y40) in Yunnan were taken. Except for the different origins, all samples were taken from the same parts of the plant. The samples were of the same grade, planting environment, climatic characteristics, cultivation, drying, fermentation technology and posttreatment process, as shown in **Figure 1** and **Table 1**. The appearance characteristics of the tobacco leaves were relatively similar, except for the size specifications. The colour is mainly brownish red, and the overall lustre is darker.

**Table 1:** Information of cigar tobacco samples.

Sample Type	Planting place					Climatic phase dates, seasons						
	Longitude and latitude	Altitude (m)	Annual rainfall (mm)	Average annual temperature (°C)	Average annual sunshine (h)	Transplanting stage	Establishment stage	Rosette stage	Floral initiation stage	Topping stage	Sucker control stage	Harvesting stage
BS-Y1-1	25° 08' 30.22'' N, 99° 11' 54.81'' E	817	1480	21.9	2329.7	2.15, spring	2.20, spring	3.5, spring	3.25, spring	4.1, spring	4.5, spring	4.10, spring
CX-Y38	25° 02' 44.44'' N, 101° 31' 41.89'' E	1135.8	600-700	21.9	2670	5.6-5.10, summers	5.17, summers	5.12-5.15, summers	5.28-5.30, summers	5.28-5.30, summers	6.25-7.26, summers	6.25-7.26, summers
DL-Y39	25° 36' 23.57'' N, 100° 16' 3.39'' E	1550	876.3	17.3	2200	5.12, summers	5.20, summers	6.2, summers	7.1, autumn	7.5, autumn	7.12, autumn	7.10, autumn
DH-Y36	24° 25' 59.33'' N, 98° 35' 8.24'' E	742.5	1500	19.2	2318.7	2.11, spring	2.13, spring	3.1, spring	4.1, spring	4.6, spring	4.11, spring	4.10, spring
LC-Y1	23° 35' 12'' N, 99° 8' 22'' E	457	1270.9	23.7	2158	2.26, spring	3.6, spring	3.25, spring	4.11, spring	4.12, spring	4.19, spring	4.19, spring
PE-Y2	22° 43' 06.28'' N, 101° 55' 84.55'' E;	813	1632	20.8	1941.1	2.20, spring	2.25, spring	3.15, spring	3.30, spring	4.10, spring	4.18, spring	4.15, spring
QJ-Y38	103° 47' 46.64'' N, 25° 29' 27.12'' E	1177.2	1055	15.9	1639.9	4.26, spring	5.5, summers	5.12, summers	6.22, summers	6.25, summers	6.28, summers	7.7, summers
WS-Y38	23° 20' 57.87'' N, 104° 15' 59.89'' E	470	1840	21.3	1804	4.4, spring	4.7, spring	4.23, spring	5.14, summers	5.20, summers	5.23, summers	5.27, summers
YX-Y6	23° 59' 24'' N, 101° 43' 48'' E	560	877	23.7	2321.2	5.12, summers	5.15, summers	6.4, summers	6.26, summers	6.26, summers	6.28-8.3, summers	7.1, summers
ZT-Y40	28° 57' 02'' N, 103° 90' 44'' E,	858	1230	18	1311.6	4.9, spring	4.18, spring	5.7, summers	6.6, summers	6.9, summers	6.16, summers	6.14, summers

### 2.2 Equipment and reagents

In the GC-IMS experiments, the instruments were used for testing with a Flavour Spec1H1-00053 GC-IMS coupler from G.A.S, Germany. In the LC-MS experiments, the instruments were an HPLC Ultim3000 and an ultra-high-resolution mass spectrometer (MS) Orbitrap Exploris 480 from Thermo Scientific, a centrifuge 5424R from Eppendorf, and a precision balance model MS105DU from Mettler Toledo.

The reagents used during the LC-MS experiments were methanol (≥95%), formic acid (≥95%) and acetonitrile (≥95%), all from Thermo Scientific.

### 2.3 Sample preparation

#### 2.3.1 GC-IMS

Ten samples of cigar tobacco leaves were crushed into powder. Three parallels were taken for each sample and placed in bottles, which were sealed and stored at room temperature. The cigar tobacco leaf sample (0.5 g) was weighed and placed in a 20 mL headspace bottle. The sample was incubated at 80°C and 500 rpm for 30 min before injection (Maurya et al., 2018).

#### 2.3.2 LC-MS

Sample pretreatment was the same as in Section 1.2.1. The 200 mg cigar sample was weighed and placed in a 1.5 mL centrifugation tube, and 10 μL internal standard (10 ppm L-2-chlorophenylalanine) and 1000 μL extract solution (methanol/acetonitrile/water, 2:2:1) were added, vortexed for 1 min, and subjected to ultrasound for 30 min. The centrifuge tube was placed in a low-temperature centrifuge and centrifuged for 5 min at 4°C and 12,000 rpm. After centrifugation, the supernatant was taken and concentrated by vacuum centrifugation for 4 h. Then, 200 μL of 50% methanol solution were added, vortexed for 40 s, and sonicated for 10 min. The 200 μL sample was filtered by a filter membrane and placed into a 200 μL intubation tube for detection. Equal volume samples were taken from each experimental sample and mixed as QC samples for machine testing.

### 2.4 GC-IMS analysis

GC-IMS analysis was performed on a column (TG-WAX). The GC programme was set as follows: the injection needle temperature was 85°C, and the carrier gas was high-purity nitrogen. The non-shunt mode was selected, and the injection volume was 500 μL. The temperature of the chromatographic column was set at 60°C, and the analysis time was 35 min. The temperature of the IMS migration tube (53 mm long) was set at 45°C, and its drift gas used high-purity nitrogen. The positive-ion mode was used in the analysis process. The gas flow rate programme was as follows: drift gas flow rate of 150 mL/min and carrier gas flow rate of 2 mL/min for 0–2 min to start data acquisition; drift gas flow rate of 150 mL/min and carrier gas flow rate of 10 mL/min for 2–10 min; drift gas flow rate of 150 mL/min and carrier gas flow rate of 100 mL/min for 10–20 min; and drift gas flow rate of 150 mL/min and carrier gas flow rate of 100 mL/min for 20–30 min. Finally, data acquisition was stopped at 30 min (Han et al., 2024).

### 2.5 LC-MS analysis

LC-MS analysis was performed on a Thermo Scientific U3000 fast LC and reverse-phase column. The chromatographic separation of the target compounds was performed on a Waters HSS T3 LC column. The mobile phases were phase A (water and 0.1% formic acid) and phase B (acetonitrile and 0.1% formic acid) in positive mode and phase A (water) and phase B (acetonitrile) in negative mode. The elution gradient programme was as follows: From 0 to 0.5 min, phase B was 5% and phase A was maintained at 95%. From 0.5 to 5.5 min, phase B was increased linearly from 5% to 90%. From 5.5 to 9 min, phase B was increased from 90% to 95% and phase A was decreased to 5%. From 9 to 10.5 min, phase B was maintained at 95% and phase A was maintained at 5%. From 10.5 to 10.6 min, phase B decreased linearly from 95% to 5% and phase A increased to 95%. From 10.6 to 12 min, phase B was maintained at 5% and phase A was maintained at 95%. The mobile phase flow rate was 0.4 mL/min, and the sample injection volume was 1 μL. The column temperature was set at 50°C.

The MS conditions were set as follows: a quadrupole-electrostatic orbitrap MS (Orbitrap Exploris 480) equipped with a thermospray ion source was used for MS analysis. Ion source: 3500 V (positive ions) and 2500 V (negative ions), sheath gas of 50 Arb, auxiliary gas flow rate of 10 Arb, and sweep gas flow rate of 1 Arb. Ion transfer tube temperature of 325°C, evaporator temperature of 350°C, full scanning. Orbital trap resolution of 60,000, scanning range of 67–1000 m/z, radiofrequency lens of 40%, maximal injection time 100 ms, intensity threshold 5.0e3 ddMS². Separation window 1 m/z. Collision energy type: normalised, collision energy 20/40/60 in HCD mode, orbital resolution: 30,000, maximum injection time 54 ms, microscopic scan was set as 1.

### 2.6 Statistical analysis

GC-IMS data were analysed using the VOCal analysis software of the equipment to view the spectra, and each point in the graph represents a volatile organic compound (VOC); the standard curve can be quantitatively analysed after the establishment of the standard curve; the built-in NIST and IMS databases of GC-IMS Library Search were used to perform two-dimensional (2D) characterisation of the characteristic peaks. The Reporter plug-in was used to compare the differences in spectra between samples [2D top view and three-dimensional (3D) spectra]; the Gallery Plot plug-in was used to construct the fingerprints of volatile compounds to visually and quantitatively analyse the differences; the experiments were conducted thrice in parallel; and the relative contents of volatile compounds were obtained by peak area normalisation and expressed in terms of mean ± standard deviation. Analysis of variance and *post hoc* tests were performed using SPSS 27.0. Origin 2021 was used to draw the heatmap of similarity correlation of fingerprint profiles; SIMCA 14.1 was used to carry out partial least squares discriminant analysis (PLS-DA). The R language package and TB tools were used to carry out principal component analysis (PCA), hierarchical clustering analysis, plotting of clustering heatmaps and establishment of a support vector machine model (Joshi et al., 2013; Maurya et al., 2022, 2023; Liu et al., 2024a).

LC-MS raw data were processed with the metabolomics processing software Compound Discoverer (Thermo Scientific) for baseline filtering, peak identification, integration, retention time correction, peak alignment, etc. Finally, a data matrix of retention time, mass-to-charge ratio and peak intensity was obtained. The main databases were the Thermo Scientific database, public database, self-built database, etc. The final data matrix for subsequent analysis was obtained.

## 3 Results and discussion

### 3.1 Flavour profile of cigar tobacco leaves by GC-IMS

**Figure 2:**
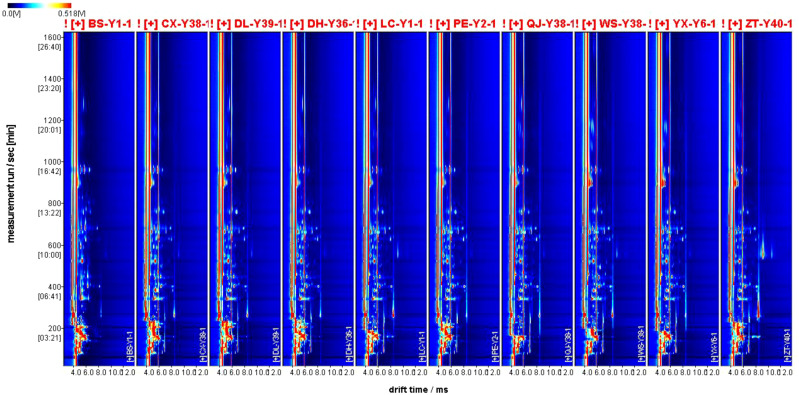
2D spectra of flavour compounds of cigar tobacco leaves from different regions.

**Figure 3:**
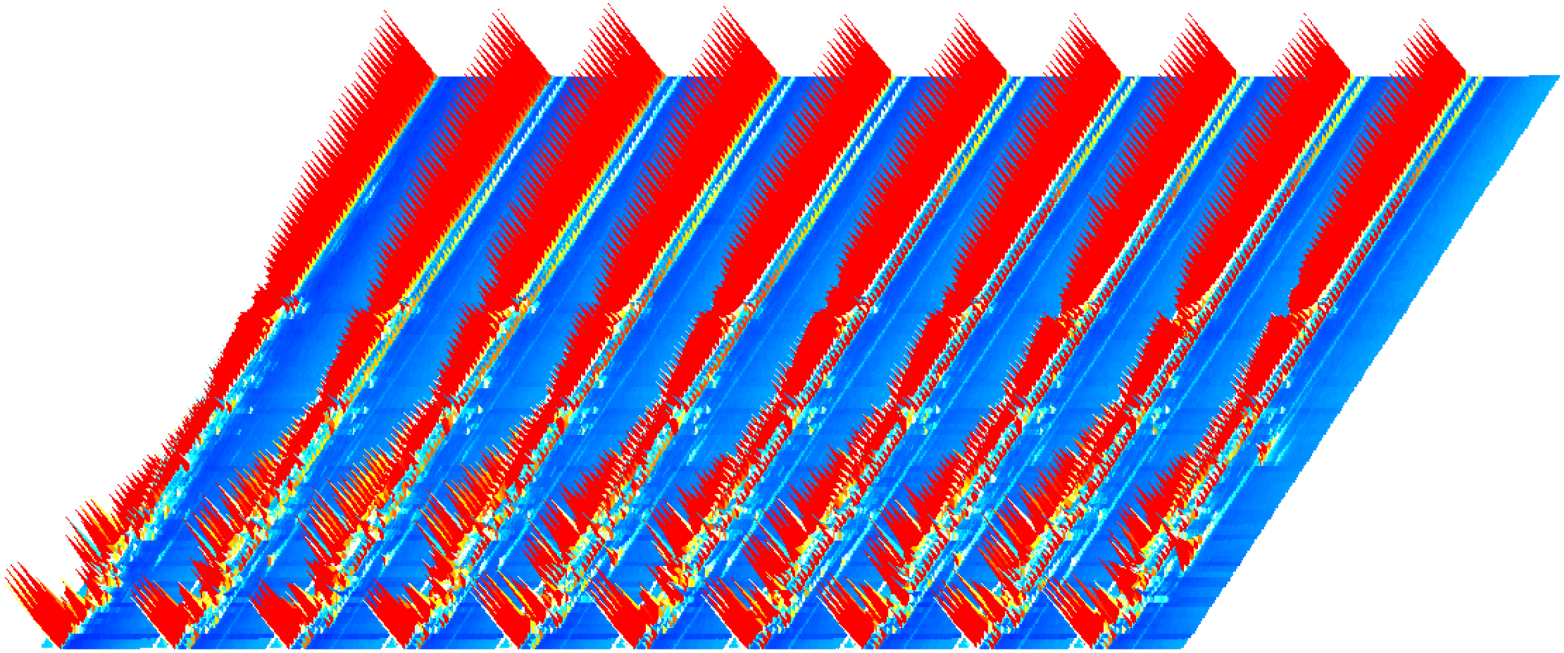
GC-IMS 3D spectra of cigar tobacco leaves from different regions.

There was little difference in the appearance of the tobacco as shown in **Figure 1**. The dark brown colour of the cigar is created by fermentation, which shifts the colour from raw green to the colour in the picture. Commonly, tobacco fermentation involves enzymes, microorganisms, and oxidation, and is a key process in the production of aromas. These aromas determine the quality of the tobacco including Maillard reaction products, carotenoids, cembranes, ladanums, glycosides, etc. The aroma of the tobacco is the result of the fermentation process (Li et al., 2022). The volatile compound results and feature region for each cigar leaf sample are shown as **Figure 2**. A total of 131 substances were detected via GC-IMS, with 109 substances characterised and 23 remaining uncharacterised (**Table 2**). In **Figure 3**, each peak represents a distinct flavour component. The intensity of the red peaks indicates the strength of the component signals. The low intensity of the red peaks indicates weak signals, which correspond to a low content of the component. Conversely, the high intensity of the red peaks indicates strong signals, which correspond to a high component content. **Figure 2** illustrates the variations in flavour profiles observed among cigar tobaccos from different geographical origins. In **Figure 2**, the red vertical line denotes the reactive ion peak. Each bright spot on either side of the peak represents a flavour component. The colour and area of the bright spot can be used to indicate the content of the flavour component. The darker the colour and the larger the area of the bright spot, the higher the content of the component. The red colour represents a higher content of the corresponding component, while the white colour represents a lower content of the corresponding component. The figure allows for a visual comparison of the flavour composition of cigar tobacco samples from 10 different origins. In **Figures 2**, **3**, the flavour components of cigar tobacco leaves from 10 distinct origins can be effectively discerned and differentiated through GC-IMS technology. Although there are discernible variations in the composition of flavour components among different *Chenopodium* album origins, the contents of the same flavour components exhibit notable discrepancies, displaying unique spectral characteristics. In order to clearly compare the specific differential substances in the 10 tobacco samples, all peaks are selected below for fingerprinting comparisons.

**Table 2:** Peak volumes of volatile compounds in cigar tobacco from 10 regions detected by GC-IMS.

No.	CAS	Compounds	BS-Y1-1	CX-Y38	DL-Y39	DH-Y36	LC-Y1	PE-Y2	QJ-Y38	WS-Y38	YX-Y6	ZT-Y40
1	122-78-1	Phenylacetaldehyde	1299.46 ± 475.55	286.13 ± 17.27	652.83 ± 14.58	674.19 ± 47.26	357.45 ± 65.06	393.62 ± 11.68	524.52 ± 64.86	223.4 ± 7.92	185.81 ± 5.91	278.37 ± 39.02
2	79-31-2	2-Methylpropanoic acid	455.75 ± 313.55	106.92 ± 18.18	103.78 ± 6.52	130.24 ± 5.62	148.35 ± 29.81	99.49 ± 32.84	122.45 ± 2.52	184.89 ± 26.79	133.6 ± 26.77	104.11 ± 8.04
3	100-52-7	Benzaldehyde-M	1199.07 ± 362.15	573.42 ± 47.77	714.14 ± 33.17	392.62 ± 40.14	185.3 ± 124.73	185.06 ± 11.52	257.29 ± 69.7	153.84 ± 71.58	102.03 ± 33.21	177.29 ± 23.97
4	18138-04-0	2,3-Diethyl-6-methylpyrazine	3145.18 ± 1611.72	1085.56 ± 125.88	1139.6 ± 104.75	747.72 ± 45.37	598.12 ± 78.29	548.91 ± 9.36	612.9 ± 6.77	587.43 ± 13.38	540.57 ± 39.39	540.68 ± 15.17
5	100-52-7	Benzaldehyde-D	758.03 ± 399.21	179.66 ± 18.55	296.44 ± 31.14	84.34 ± 17.6	42.96 ± 29.04	35.66 ± 2.75	64.03 ± 23.61	38.09 ± 9.84	27.25 ± 6.10	36.88 ± 2.35
6	64-19-7	Acetic acid-M	1393.4 ± 218.73	1167.96 ± 42.9	1103.03 ± 85.75	1468.86 ± 94.19	1247.75 ± 538.81	765.15 ± 75.09	783.34 ± 174.36	1168.51 ± 315.85	998.76 ± 246.97	961.54 ± 339.58
7	3268-49-3	Methional	633.42 ± 185.92	289.95 ± 24.24	244.94 ± 18.35	383.64 ± 11.26	374.18 ± 231.77	167.89 ± 30.47	225.3 ± 70.9	404.21 ± 157.83	327.9 ± 125.11	262.2 ± 125.88
8	95-93-2	1,2,4,5-Tetramethylbenzene	313.68 ± 62.75	74.02 ± 6.33	99.25 ± 4.16	111.63 ± 8.3	52.91 ± 25.79	47.28 ± 2.21	32.83 ± 8.49	21.93 ± 8.37	33.12 ± 7.90	30.58 ± 11.58
9	64-19-7	Acetic acid-D	163.76 ± 47.35	82.54 ± 10.82	74.21 ± 14.61	208.91 ± 21.27	341.4 ± 185.05	113.07 ± 29.12	94.53 ± 27.58	498.76 ± 101.04	222.29 ± 82.08	152.88 ± 72.4
10	928-96-1	(Z)-Hex-3-enol-M	438.41 ± 11.72	387.5 ± 1.24	242.5 ± 1.45	292.32 ± 20.64	162.38 ± 55.68	160.75 ± 7.81	327.57 ± 53.96	277.55 ± 68.98	198.62 ± 42.81	174.23 ± 70.03
11	928-96-1	(Z)-Hex-3-enol-D	398.43 ± 30.81	472.93 ± 14.72	806.96 ± 13.05	591.02 ± 27	346.07 ± 97.32	404.67 ± 25.62	291.3 ± 33.47	236.46 ± 54.32	427.98 ± 101.85	356.16 ± 128.87
12	110-93-0	6-Methylhept-5-en-2-one	1408.68 ± 87.01	1419.07 ± 13.18	1713.47 ± 39.26	1408.3 ± 51.93	1179.44 ± 144.49	1536.69 ± 46.02	1200.39 ± 78.25	1097.96 ± 80.28	926.6 ± 64.01	1110.18 ± 154.68
13	57-06-7	Allyl isothiocyanate	575.01 ± 101.35	1102.89 ± 136.11	911.9 ± 103.86	1013.23 ± 17.93	1042.54 ± 58.18	994.92 ± 19.29	994.42 ± 9.83	1137.58 ± 53.86	1147.02 ± 80.1	847.07 ± 253.24
14	68-12-2	N,N-dimethylformamide	301.91 ± 10.55	533.29 ± 115.88	335.66 ± 48.72	632.85 ± 54.14	787.95 ± 134.23	712.03 ± 33.89	525.53 ± 23.7	729.27 ± 52.34	582.06 ± 71.62	282.36 ± 102.65
15	97-64-3	Ethyl lactate-D	87.43 ± 32.71	288.87 ± 35.67	262.09 ± 10.92	355.98 ± 19.48	561.63 ± 109.52	686.54 ± 18.03	514.03 ± 48.58	627.6 ± 83.63	585.89 ± 82.75	384.25 ± 47.38
16	589-35-5	3-Methyl-1-pentanol	410.88 ± 184.31	1686.94 ± 248.59	1443.56 ± 109.95	1996.84 ± 104.61	2529.49 ± 149.7	2720.63 ± 35.3	2142.59 ± 130.55	2570.51 ± 175.13	2317 ± 158.85	1505.8 ± 182.99
17	unidentified	Unknown 1	916.64 ± 116.23	601.01 ± 17.1	782.16 ± 12.55	599.15 ± 35.97	562.34 ± 26.56	684.02 ± 14.45	707.11 ± 36.19	669.86 ± 22.54	631.43 ± 63.75	717.26 ± 98.87
18	16409-43-1	Rose oxide	389.05 ± 66.21	719.77 ± 108.78	1286.87 ± 152.55	1358.57 ± 73.02	1368.81 ± 34.35	1423.13 ± 38.77	1239.49 ± 38.73	812.69 ± 24.66	1284.4 ± 40.68	901.81 ± 68.81
19	97-64-3	Ethyl lactate-M	35.84 ± 8.25	17.97 ± 1.73	23.02 ± 0.14	64.85 ± 9.05	40.32 ± 8.14	43.24 ± 4.27	55.94 ± 12.64	21.62 ± 4.04	21.18 ± 3.78	53.09 ± 7.26
20	123-32-0	2,5-Dimethylpyrazine	185.93 ± 9.74	196.3 ± 6.20	126.93 ± 28.52	313.01 ± 37.73	188.76 ± 36.86	196.83 ± 30.03	95.62 ± 22.63	141.07 ± 5.41	142.46 ± 12.25	159.73 ± 31.28
21	18829-55-5	(E)-2-heptenal	101.23 ± 5.75	422.01 ± 24.74	83.89 ± 6.77	350.13 ± 7.05	80.22 ± 12.13	89.57 ± 8.22	103.16 ± 4.38	234.18 ± 15.91	145.32 ± 17.61	110.82 ± 9.27
22	134-20-3	Methyl anthranilate	38.50 ± 8.38	294.22 ± 39	79.42 ± 2.23	347.8 ± 5.61	98.73 ± 4.64	133.58 ± 8.96	139.19 ± 1.97	266.05 ± 4.93	160.55 ± 12.58	101.8 ± 10.9
23	108-94-1	Cyclohexanone-M	940.37 ± 306.04	656.53 ± 25.85	399.19 ± 21.57	612.22 ± 14.38	486.54 ± 45.48	285.15 ± 11.43	648.21 ± 13.2	536.25 ± 27.85	771.9 ± 76.54	373.26 ± 46.03
24	89-83-8	Thymol	266.33 ± 42.86	402.71 ± 23.96	310.17 ± 17.94	289.93 ± 7.26	324.01 ± 17.22	373.89 ± 14	304.55 ± 12.24	240.02 ± 16.09	212.55 ± 10.89	222.98 ± 23.83
25	124-13-0	Octanal	74.08 ± 15.69	97.68 ± 3.20	107.15 ± 11.7	112.5 ± 3.01	91.31 ± 5.18	107.16 ± 5.21	103.18 ± 7.25	93.93 ± 7.29	124.69 ± 12.5	149.25 ± 14.55
26	108-94-1	Cyclohexanone-D	75.98 ± 7.84	111.4 ± 1.56	225.94 ± 26.37	128.83 ± 3.45	97.8 ± 10.07	137.66 ± 18.53	90.92 ± 3.64	81.56 ± 4.04	84.24 ± 1.30	97.98 ± 6.50
27	4312-99-6	1-Octen-3-one	26.79 ± 9.62	108.04 ± 16.54	138.07 ± 12.09	172.33 ± 4.68	260.73 ± 28.98	230.22 ± 15.12	430.58 ± 14.35	158.73 ± 7.04	189.1 ± 4.11	151.4 ± 7.02
28	541-58-2	2,4-Dimethylthiazole	249.02 ± 74.48	243.81 ± 15.8	290.65 ± 24.18	361.66 ± 1.88	414.68 ± 21.18	316.35 ± 11.99	573.77 ± 29.84	235.2 ± 19.62	274.03 ± 20.02	235.47 ± 24.62
29	116-09-6	1-Hydroxypropan-2-one	248.06 ± 15.81	1075.23 ± 31.44	393.84 ± 8.17	216.34 ± 1.87	208.97 ± 2.82	284.81 ± 20	240.72 ± 15.25	463.27 ± 10.03	500.66 ± 27.86	301.76 ± 24.97
30	108-48-5	2,6-Dimethylpyridine	413.07 ± 118	634.84 ± 12.33	790.49 ± 25.21	1314.01 ± 94.55	1235.81 ± 16.01	1223.56 ± 76.98	994.37 ± 64.87	814.47 ± 39.07	739.22 ± 52.06	804.58 ± 59.52
31	626-89-1	4-Methyl-1-pentanol	13.84 ± 1.88	72.26 ± 4.85	16.00 ± 1.35	67.00 ± 2.36	37.74 ± 9.01	41.88 ± 1.93	59.60 ± 2.43	97.86 ± 7.20	69.38 ± 5.60	40.63 ± 4.47
32	6028-61-1	Dipropyl trisulfide	397.06 ± 18.47	351.12 ± 12.83	553.43 ± 27.86	481.02 ± 74.1	576 ± 26.68	541.05 ± 36.19	503.37 ± 16.42	447.49 ± 13.8	495.86 ± 33.16	521.2 ± 27.00
33	543-49-7	2-Heptanol	113.83 ± 19.08	205.11 ± 34.18	156.95 ± 15.08	499.75 ± 28.34	445.05 ± 82.03	403.71 ± 4.18	305.24 ± 8.16	301.97 ± 27.7	243.66 ± 33.88	179.92 ± 63.58
34	142-92-7	Hexyl acetate	15.92 ± 6.23	27.94 ± 2.18	47.07 ± 2.36	129.47 ± 5.89	102.07 ± 11.86	127.58 ± 18.71	72.76 ± 6.48	67.69 ± 3.53	35.12 ± 3.17	56.48 ± 8.17
35	109-08-0	2-Methylpyrazine	120.48 ± 43.31	385.38 ± 21.46	314.46 ± 11.14	468.47 ± 17.26	334.65 ± 18.97	392.56 ± 15.66	300.46 ± 13.45	404.06 ± 16.59	302.03 ± 21.37	276.22 ± 15.96
36	99-85-4	γ-Terpinene-M	1267.04 ± 63.58	1382.89 ± 23.06	1497.94 ± 34.44	1301.45 ± 37.28	1311.14 ± 49.72	1133.86 ± 36.59	1150.53 ± 9.05	1082.4 ± 27.9	1150.54 ± 94.61	1114.38 ± 51.87
37	100-42-5	Styrene	151.62 ± 4.13	215.74 ± 4.05	200.64 ± 8.15	299.06 ± 7.54	179.82 ± 7.08	237.17 ± 24.1	150.38 ± 4.1	124.97 ± 5.05	153.99 ± 7.79	145.57 ± 8.4
38	763-32-6	3-Methyl-3-buten-1-ol	20.39 ± 0.98	142.05 ± 5.97	89.41 ± 5.85	39.12 ± 2.05	24.03 ± 1.05	64.61 ± 7.27	21.06 ± 1.43	261.1 ± 15.79	25.67 ± 0.62	220.5 ± 2.61
39	99-85-4	γ-Terpinene-D	34.21 ± 13.57	268.71 ± 62.48	246.26 ± 14.77	423.17 ± 27.64	340.99 ± 21.84	480.34 ± 14	352.27 ± 19.79	418.55 ± 23.39	395.09 ± 11.6	291.4 ± 8.46
40	290-37-9	Pyrazine	157.71 ± 56.75	517.64 ± 12.07	596.62 ± 16.7	808.9 ± 22.73	715.15 ± 25.58	847.17 ± 17.69	784.52 ± 10.09	632.65 ± 8.69	644.45 ± 40.92	414.19 ± 9.08
41	110-86-1	Pyridine-M	1914.61 ± 274.12	1896.3 ± 56.71	1930.23 ± 21.57	1869.5 ± 37.17	1790.18 ± 62.9	1754.3 ± 36.65	1644.8 ± 31.33	1579.78 ± 42.29	1563.32 ± 47.94	1785.22 ± 17.12
42	unidentified	Unknown 2	295.87 ± 40.44	583.38 ± 28.23	489.83 ± 16.32	628.5 ± 41.63	515.4 ± 21.56	397.37 ± 9.56	441.78 ± 5.68	99.42 ± 2.75	556.68 ± 22.45	176.22 ± 2.63
43	626-93-7	Hexan-2-ol	95.43 ± 10.05	68.21 ± 4.10	64.52 ± 6.08	62.82 ± 3.18	46.89 ± 1.71	66.02 ± 4.81	51.05 ± 2.45	114.42 ± 4.2	58.31 ± 6.91	112.01 ± 0.34
44	111-12-6	2-Octynoic acid, methyl ester	853.61 ± 217.97	1057.87 ± 34.32	1284.71 ± 50.04	1047.78 ± 11.66	1094.1 ± 11.27	968.24 ± 16.19	845.75 ± 22.82	2034.49 ± 66.5	908.14 ± 18.96	1028.77 ± 11.17
45	unidentified	Unknown 3	14.37 ± 2.40	83.87 ± 15.73	77.42 ± 8.58	98.43 ± 3.29	134.84 ± 15.48	133.01 ± 11.98	215.76 ± 2.49	316.58 ± 19.28	175.25 ± 10.51	148.88 ± 6.08
46	110-86-1	Pyridine-D	1285.27 ± 360.64	1296.92 ± 39.22	1442.34 ± 69.42	1404.2 ± 20.15	1327.94 ± 66.23	1452.38 ± 29.09	1310.14 ± 37.26	1415.15 ± 46.73	1039.48 ± 47.57	1372.51 ± 41.01
47	unidentified	Unknown 4	523.5 ± 130.8	489.44 ± 33.54	497.16 ± 29.79	628.47 ± 52.91	625.15 ± 15.17	549.88 ± 21.03	572.05 ± 10.82	520.28 ± 17.49	626.93 ± 60.52	563.83 ± 38.76
48	unidentified	Unknown 5	24.88 ± 13.2	217.66 ± 44.9	242.01 ± 6.75	258.52 ± 14.29	304.67 ± 29.85	359.02 ± 14.72	344.97 ± 20.58	341.38 ± 17.95	358.98 ± 15.33	344.33 ± 23.67
49	105-68-0	3-Methylbutyl propanoate	506.71 ± 110.08	404.62 ± 24.86	559.08 ± 40.16	1055.48 ± 34.76	906.95 ± 18.54	923 ± 22.27	813.21 ± 18.71	686.52 ± 11.33	879.56 ± 30.98	760.97 ± 24.03
50	123-35-3	β-Myrcene-M	613.98 ± 136.1	1722.22 ± 26.23	1086.93 ± 22.49	1010.87 ± 18.46	947.44 ± 10.39	1055.53 ± 26.21	1155.08 ± 24.85	1312.79 ± 29.02	1076.76 ± 17.95	1200.84 ± 41.65
51	unidentified	Unknown 6	19.30 ± 3.83	302.92 ± 62.15	245.22 ± 13.08	230.77 ± 7.28	229.47 ± 12.06	311.37 ± 9.73	279.17 ± 7.18	397.47 ± 14.28	343.44 ± 11.13	306.93 ± 14.15
52	123-35-3	β-Myrcene-D	153.03 ± 6.46	311.23 ± 28.71	243.7 ± 0.36	417.86 ± 5.01	266.80 ± 15.01	249.72 ± 6.15	249.91 ± 7.69	197.10 ± 8.06	303.29 ± 11.08	222.07 ± 0.29
53	6728-26-3	(E)-2-hexenal	120.66 ± 11.78	194.9 ± 3.84	160.02 ± 3.99	272.63 ± 4.34	98.10 ± 8.32	86.31 ± 1.46	59.88 ± 2.53	106.90 ± 3.38	113.66 ± 3.00	107.10 ± 4.64
54	110-43-0	2-Heptanone-M	222.73 ± 13.36	95.61 ± 1.06	99.40 ± 0.69	100.86 ± 1.43	112.83 ± 7.93	112.71 ± 7.87	143.30 ± 3.25	147.79 ± 7.18	130.34 ± 5.82	103.66 ± 3.75
55	470-82-6	1,8-Cineole	86.14 ± 5.91	109.86 ± 19.11	100.04 ± 5.00	108.25 ± 23.07	116.38 ± 13.71	148.37 ± 6.54	142.99 ± 24.20	106.58 ± 4.07	125.40 ± 29.34	152.02 ± 7.18
56	unidentified	Unknown 7	417.77 ± 13.68	370.95 ± 8.13	418.57 ± 12.42	377.29 ± 18.63	331.46 ± 28.12	341.63 ± 37.23	333.14 ± 14.61	275.29 ± 8.00	382.1 ± 18.05	310.76 ± 13.85
57	111-71-7	Heptanal	69.35 ± 14.98	212.06 ± 14.02	185.44 ± 0.22	248.4 ± 9.63	186.97 ± 6.46	146.93 ± 4.19	146.1 ± 2.39	47.07 ± 4.73	230.87 ± 1.25	52.01 ± 1.41
58	109-21-7	Butyl butanoate	100.43 ± 38.09	140.34 ± 3.62	219.00 ± 8.56	148.6 ± 4.59	155.65 ± 3.12	137.63 ± 9.39	105.28 ± 8.51	320.03 ± 14.07	120.23 ± 5.35	146.67 ± 8.23
59	138-86-3	Limonene	194.51 ± 2.43	741.58 ± 33.79	640.55 ± 7.26	866.81 ± 4.24	755.99 ± 28.76	632.83 ± 51.97	784.9 ± 12.2	359.04 ± 6.98	631.53 ± 31.48	389.58 ± 20.06
60	103-65-1	Propylbenzene	134.5 ± 21.86	94.07 ± 7.89	106.45 ± 4.51	150.35 ± 1.47	141.27 ± 17.39	155.86 ± 14.01	138.12 ± 12.9	137.97 ± 2.4	123.7 ± 4.09	130.44 ± 2.9
61	110-43-0	Heptan-2-one-D	43.6 ± 11.11	106.93 ± 11.15	135.03 ± 2.12	169.47 ± 7.95	170.71 ± 16.69	207.02 ± 9.92	189.62 ± 2.86	184.55 ± 3.31	196.49 ± 1.82	184.94 ± 10.93
62	89-49-6	Isopulegyl acetate	17.23 ± 2.66	17.38 ± 2.53	25.51 ± 2.41	38.10 ± 2.64	30.12 ± 1.89	47.20 ± 3.65	29.63 ± 1.63	104.46 ± 3.51	18.44 ± 1.86	102.66 ± 9.48
63	29926-41-8	2-Acetyl-2-thiazoline	172.84 ± 27.26	207.79 ± 13.5	204.11 ± 3.31	195.01 ± 7.97	204.05 ± 5.76	194.6 ± 9.95	190.67 ± 12.12	189.49 ± 1.17	205.93 ± 19	210.81 ± 4.85
64	unidentified	Unknown 8	676.62 ± 165.33	578.28 ± 22.61	575.71 ± 10.26	609.61 ± 31.24	586.51 ± 11.87	527.72 ± 11.95	504.82 ± 6.2	500.06 ± 9.22	515.04 ± 21.99	547.31 ± 19.4
65	100-41-4	Ethylbenzene	804 ± 39.88	823.08 ± 21.17	795.63 ± 13.18	654.32 ± 26.01	655.63 ± 32.32	666.1 ± 8.64	662.45 ± 4.99	578.18 ± 10.72	568.93 ± 12.98	643.27 ± 11.61
66	6032-29-7	2-Pentanol	588.84 ± 82.71	548.12 ± 16.99	541.49 ± 12.21	604.09 ± 13.03	474.53 ± 27.93	617.52 ± 16.6	509.71 ± 24.13	591.39 ± 6.03	459.56 ± 24.13	592.73 ± 46.52
67	624-92-0	Dimethyl disulfide	385.56 ± 80.93	327.38 ± 17.54	233 ± 13.07	255.45 ± 8.9	302.09 ± 68.04	220.19 ± 51.04	402.12 ± 92.27	545.15 ± 87.41	287.69 ± 50.49	384.29 ± 121.6
68	108-88-3	Toluene	1919.81 ± 211.49	1864.76 ± 30.56	1682.34 ± 13.19	1615.7 ± 9.1	1580.47 ± 37.85	1536.77 ± 7.93	1411.98 ± 4.66	1567.45 ± 21.47	1597.03 ± 13.25	1854.59 ± 61.07
69	1629-58-9	1-Penten-3-one	788.59 ± 93.54	284.51 ± 59.25	247.65 ± 2.00	188.29 ± 3.53	153.88 ± 11.82	101.31 ± 1.79	111.36 ± 6.29	129.56 ± 2.04	179.04 ± 7.26	214.03 ± 14.86
70	71-23-8	1-Propanol	84.85 ± 11.3	60.92 ± 4.95	40.61 ± 2.73	85.37 ± 5.92	85.05 ± 2.68	95.96 ± 4.54	128.36 ± 9.63	72.52 ± 1.61	87.48 ± 0.85	84.87 ± 3.19
71	591-78-6	Hexan-2-one	499.52 ± 69.04	333.62 ± 9.31	249.24 ± 7.3	463.98 ± 15.8	367.99 ± 10.57	208.9 ± 13.59	240.21 ± 3.81	219.31 ± 17.15	291.16 ± 30.86	227.9 ± 17.58
72	7452-79-1	2-Methyl butanoic acid ethyl ester	494.73 ± 59.56	168.76 ± 30.42	131.21 ± 8.39	895.72 ± 35.03	472.23 ± 30.94	1654.83 ± 44.67	846.27 ± 29	389.34 ± 22.18	220.52 ± 7.59	372.85 ± 34.38
73	110-19-0	Isobutyl acetate	745.07 ± 181.3	892.37 ± 56.68	835.94 ± 85.09	867.04 ± 28.1	746.62 ± 40.05	861.5 ± 41.15	514.39 ± 5.77	676.89 ± 23.62	736.17 ± 7.17	732.01 ± 27.19
74	123-75-1	Pyrrolidine	761.58 ± 68.74	370.8 ± 71.94	402.36 ± 26.77	524.41 ± 35.11	296.86 ± 11.35	230.39 ± 1.91	165.7 ± 14.9	193.64 ± 6.19	244.65 ± 9.75	256.34 ± 17.51
75	107-87-9	2-Pentanone	524.09 ± 17.13	562.9 ± 8.05	376.87 ± 13.59	668.72 ± 22.22	268.97 ± 19.94	398.6 ± 10.97	211.53 ± 2.48	252.47 ± 5.36	471.1 ± 18.2	327.97 ± 11.3
76	78-92-2	Butan-2-ol	286.81 ± 40.99	763.58 ± 68.75	770.67 ± 23.22	727.62 ± 26.12	828.92 ± 41.17	967.58 ± 32.58	881.64 ± 44.66	825.47 ± 48.57	915.95 ± 54.65	765.66 ± 80.81
77	unidentified	Unknown 9	90.03 ± 20.88	576.38 ± 100.55	616.65 ± 10.81	503.44 ± 10.51	716.54 ± 77.51	840.16 ± 27.36	715.92 ± 52.78	851.85 ± 42.68	780.18 ± 24.14	694.88 ± 55.62
78	105-54-4	Ethyl butanoate	73.33 ± 77.65	347.79 ± 16.34	342.68 ± 5.34	750.2 ± 29.32	637.94 ± 20.81	974.25 ± 34.5	764.78 ± 32.41	601.62 ± 2.6	514.9 ± 16.96	559.44 ± 53.54
79	unidentified	Unknown 10	202.5 ± 11.78	47.57 ± 3.10	107.85 ± 3.58	108.86 ± 1.55	273.15 ± 5.72	222.78 ± 17.16	225.64 ± 11.54	112.8 ± 4.07	32.89 ± 1.04	102.68 ± 8.18
80	78-59-1	Isophorone	162.19 ± 45.69	68.20 ± 18.43	67.18 ± 5.89	87.92 ± 4.66	62.97 ± 5.11	49.90 ± 0.86	44.72 ± 3.69	37.18 ± 5.19	52.36 ± 4.57	55.28 ± 4.59
81	unidentified	Unknown 11	70.65 ± 7.55	117.37 ± 9.39	100.62 ± 8.41	116.89 ± 4.46	80.74 ± 1.80	133.03 ± 4.1	76.09 ± 1.91	96.83 ± 4.73	95.11 ± 3.09	83.30 ± 2.58
82	unidentified	Unknown 12	34.97 ± 3.24	47.78 ± 5.29	31.20 ± 1.08	75.12 ± 1.28	44.55 ± 4.68	54.89 ± 4.32	33.24 ± 1.83	83.17 ± 2.65	53.57 ± 1.95	47.80 ± 4.42
83	123-86-4	Butyl acetate	85.21 ± 2.58	58.02 ± 4.31	58.52 ± 2.83	125.32 ± 5.4	104.96 ± 3.82	92.71 ± 2.16	43.59 ± 5.98	50.93 ± 1.98	71.60 ± 3.06	74.93 ± 4.60
84	66-25-1	Hexanal	100.24 ± 10.92	114.76 ± 2.91	111.32 ± 4.59	157.18 ± 1.95	93.68 ± 4.66	130.62 ± 5.41	127.24 ± 9.09	87.63 ± 2.08	110.06 ± 1.89	69.07 ± 2.61
85	98-82-8	(1-Methylethyl) benzene	468.45 ± 66.16	80.69 ± 5.55	123.4 ± 4.06	135.56 ± 8.93	94.58 ± 18.71	93.29 ± 18.1	62.15 ± 2.19	52.66 ± 3.06	49.68 ± 3.84	68.80 ± 4.45
86	unidentified	Unknown 13	15.23 ± 4.35	24.49 ± 2.70	13.59 ± 2.40	60.89 ± 5.32	10.88 ± 1.24	18.40 ± 3.24	7.03 ± 0.33	95.79 ± 6.83	8.89 ± 0.60	32.93 ± 5.68
87	556-24-1	Methyl 3-methylbutanoate	87.01 ± 14.19	87.31 ± 1.98	101.61 ± 7.7	121.95 ± 1.84	103.62 ± 2.63	100.98 ± 0.94	96.52 ± 3.87	87.15 ± 3.06	104.39 ± 4.05	78.66 ± 1.23
88	unidentified	Unknown 14	65.67 ± 3.10	106.72 ± 7.07	98.84 ± 2.91	94.22 ± 4.42	75.91 ± 6.00	88.46 ± 4.06	86.69 ± 2.85	67.85 ± 1.27	92.70 ± 5.39	82.28 ± 5.46
89	105-66-8	Butanoic acid propyl ester	5.58 ± 1.33	40.61 ± 9.51	39.88 ± 5.62	53.62 ± 4.23	50.28 ± 11.73	81.44 ± 1.77	50.43 ± 5.45	66.57 ± 2.79	75.19 ± 4.25	53.24 ± 6.27
90	1577-18-0	3-Hexenoic acid, (E)	114.5 ± 34.42	26.02 ± 2.14	24.96 ± 2.52	412.05 ± 44.75	210.58 ± 6.54	126.00 ± 2.72	93.46 ± 2.61	15.96 ± 2.23	146.63 ± 1.48	27.66 ± 1.31
91	110-62-3	Pentanal	232.72 ± 11.33	183.31 ± 25.25	116.65 ± 6.93	376.05 ± 49.16	96.77 ± 8.68	87.15 ± 3.02	54.04 ± 2.27	63.33 ± 3.41	125.44 ± 6.44	76.11 ± 6.04
92	590-86-3	3-Methylbutanal-D	1421.82 ± 79.81	1141.08 ± 63.73	1021.48 ± 218.18	712.12 ± 18.71	699.7 ± 24.12	524.08 ± 59.42	383.55 ± 14.07	1001.36 ± 23.08	559.26 ± 20.92	652.6 ± 24.2
93	78-93-3	Butan-2-one	334.38 ± 150.91	404.85 ± 22.98	379.18 ± 36.37	196.5 ± 6.18	66.07 ± 1.89	375.45 ± 7.58	68.37 ± 3.35	2268.38 ± 30.43	113.08 ± 2.27	584.17 ± 39.72
94	unidentified	Unknown 15	23.60 ± 3.60	321.78 ± 2.14	237.43 ± 44.90	250.86 ± 13.78	16.81 ± 1.65	267.46 ± 58.68	18.66 ± 0.58	25.71 ± 0.38	15.16 ± 1.72	184.14 ± 6.57
95	3208-16-0	2-Ethylfuran	46.88 ± 11.90	754.58 ± 10.97	692.09 ± 101.82	470.15 ± 9.48	35.04 ± 0.54	477.27 ± 166.57	34.06 ± 1.16	55.20 ± 0.69	44.44 ± 4.41	515.52 ± 5.85
96	141-78-6	Ethyl acetate	784.39 ± 920.19	2244.21 ± 91.02	2148.88 ± 48.56	1848.27 ± 19.11	398.44 ± 24.48	2502.44 ± 77.66	603.41 ± 15.61	358.58 ± 19.68	530.44 ± 23.64	3307.82 ± 81.42
97	123-72-8	Butanal	2680.32 ± 613.5	2679.7 ± 98.28	2571.32 ± 79.67	2534.46 ± 48.17	2522.43 ± 32.8	2805.84 ± 137.93	737.64 ± 37.04	2714.05 ± 48.57	1900.71 ± 34.32	2688.96 ± 33.69
98	108-21-4	1-Methylethyl acetate	5423.19 ± 970.11	3943.08 ± 51.41	5369.85 ± 61.36	5070.72 ± 62.77	6408.02 ± 74.05	3321.58 ± 164.47	5190.14 ± 15.04	5738.26 ± 133.6	6327.29 ± 148.39	6072.24 ± 349.91
99	105-57-7	Diethyl acetal	5368.88 ± 633.23	3892.88 ± 120.84	4347.54 ± 67.92	4453.13 ± 76.33	4169.29 ± 256.32	3574.26 ± 59.32	1547.75 ± 17.38	3968.34 ± 72.05	3290.41 ± 44.47	4242.26 ± 162.84
100	590-86-3	3-Methylbutanal-M	851.71 ± 43.2	206.5 ± 14.83	221.26 ± 25.62	194.4 ± 2.31	818.37 ± 4.92	315.79 ± 12.36	684.22 ± 4.15	771.04 ± 15.14	743.97 ± 15.55	220.43 ± 38.01
101	111-27-3	1-Hexanol	1985.11 ± 177.38	1824.93 ± 50.48	2163.45 ± 121.74	1872.93 ± 46.4	2249.21 ± 203.68	2085.61 ± 70.88	2279.25 ± 41.62	2429.87 ± 48.55	2320.62 ± 42.42	2753.97 ± 292.23
102	928-97-2	( E)-3-hexen-1-ol	67.33 ± 1.35	104.13 ± 6.83	95.61 ± 3.67	482.86 ± 3.65	119.83 ± 29.09	473.51 ± 45.68	88.38 ± 12.85	77.31 ± 15.75	413.75 ± 89.31	96.06 ± 36.82
103	127-19-5	N,N-Dimethylacetamide	257.44 ± 9.85	280.65 ± 10.16	304.68 ± 32.77	300.53 ± 9.35	186.86 ± 52.73	177.9 ± 14.96	178.11 ± 15.22	206.82 ± 31.88	175.72 ± 43.92	164.67 ± 35.96
104	629-33-4	Hexyl formate	44.29 ± 4.63	33.30 ± 2.28	41.85 ± 1.78	173.84 ± 14.8	66.68 ± 3.90	298.35 ± 25.28	59.27 ± 10.1	48.22 ± 2.55	243.5 ± 20.41	54.84 ± 13.35
105	unidentified	Unknown 16	43.75 ± 10.78	42.12 ± 1.26	54.86 ± 6.82	55.11 ± 2.64	65.59 ± 8.77	50.93 ± 3.56	65.78 ± 6.81	63.52 ± 3.49	73.89 ± 11.12	47.82 ± 6.19
106	2445-76-3	Hexyl propionate	66.89 ± 2.88	80.55 ± 5.45	53.06 ± 3.78	68.82 ± 3.22	39.91 ± 5.12	47.82 ± 2.29	64.37 ± 2.23	65.97 ± 4.30	35.98 ± 3.59	47.26 ± 6.04
107	586-62-9	α-Terpinolene	39.08 ± 3.89	43.31 ± 2.48	38.01 ± 3.99	52.71 ± 2.88	43.77 ± 1.78	40.65 ± 4.81	38.44 ± 3.11	48.60 ± 4.17	40.03 ± 7.78	35.35 ± 2.44
108	556-64-9	Thiocyanic acid methyl ester	10.64 ± 1.98	21.48 ± 2.05	24.38 ± 1.49	23.14 ± 3.70	14.70 ± 1.61	16.92 ± 1.06	15.55 ± 1.52	21.70 ± 2.77	18.03 ± 0.95	21.10 ± 1.27
109	unidentified	Unknown 17	60.66 ± 2.32	62.96 ± 2.39	56.90 ± 4.80	55.35 ± 1.83	60.90 ± 2.78	54.52 ± 4.80	109.65 ± 5.32	69.71 ± 6.05	66.77 ± 4.56	57.03 ± 5.96
110	71-41-0	1-Pentanol	96.55 ± 17.76	86.57 ± 1.16	75.91 ± 2.08	68.20 ± 4.70	43.24 ± 3.45	40.16 ± 7.09	36.78 ± 0.59	39.64 ± 1.33	56.87 ± 4.83	46.51 ± 6.06
111	unidentified	Unknown 18	106.86 ± 13.37	44.33 ± 7.33	53.08 ± 5.24	109.97 ± 1.4	67.30 ± 3.74	73.75 ± 2.40	80.02 ± 5.37	32.52 ± 2.20	38.98 ± 1.80	31.78 ± 3.18
112	623-36-9	2-Methyl-2-pentenal	218.61 ± 18.34	271.67 ± 12.28	304.34 ± 35.21	269.08 ± 23.15	293.99 ± 15.43	233.4 ± 33.06	269.92 ± 7.88	244.81 ± 9.08	238.73 ± 22.13	279.63 ± 3.89
113	105-37-3	Ethyl propanoate	376.65 ± 110.76	46.21 ± 7.01	51.31 ± 5.18	445.08 ± 22.09	72.47 ± 7.32	47.61 ± 2.90	67.05 ± 8.65	20.30 ± 0.87	57.64 ± 1.79	55.76 ± 2.36
114	108-64-5	Ethyl 3-methylbutanoate	29.72 ± 3.17	58.95 ± 4.14	27.87 ± 4.17	63.83 ± 8.28	28.23 ± 0.29	48.42 ± 2.28	34.58 ± 1.69	18.35 ± 1.35	33.41 ± 3.93	21.17 ± 1.50
115	80-56-8	α-Pinene	86.73 ± 9.26	129.29 ± 11.96	117.89 ± 14.56	135.56 ± 6.59	100.55 ± 1.5	126.97 ± 7.38	56.17 ± 3.19	99.32 ± 3.48	103.8 ± 3.62	99.82 ± 7.05
116	124-19-6	Nonanal	49.24 ± 8.07	48.68 ± 3.57	68.92 ± 8.25	75.57 ± 2.64	40.72 ± 5.99	52.91 ± 2.18	35.55 ± 1.03	31.05 ± 3.00	46.44 ± 8.41	44.49 ± 9.47
117	121-44-8	Triethylamine	269.22 ± 55.72	264.83 ± 9.44	230.66 ± 39.81	256.21 ± 3.97	152.28 ± 8.75	274.26 ± 18.25	199.44 ± 8.74	223.64 ± 3.32	133.6 ± 3.44	272.37 ± 5.22
118	unidentified	Unknown 19	283.77 ± 21	223.56 ± 16.33	216.24 ± 4.96	175.53 ± 5.16	249.27 ± 1.52	211.69 ± 9.95	269.89 ± 3.73	246.79 ± 2.73	230.78 ± 10.72	177.51 ± 44.09
119	111-66-0	1-Octene	280.73 ± 48.84	275.37 ± 29.7	283.21 ± 47.55	338.86 ± 17.98	339.05 ± 2.21	273.28 ± 16.57	245.39 ± 16.23	215.91 ± 8.2	304.53 ± 4.16	204.83 ± 19.5
120	unidentified	Unknown 20	144 ± 18.04	131.01 ± 17.5	93.99 ± 4.45	86.34 ± 4.31	211.7 ± 6.63	72.90 ± 7.06	174.5 ± 14.46	89.17 ± 1.26	271.53 ± 5.52	99.76 ± 6.81
121	78-84-2	2-Methyl propanal	306.58 ± 131.98	317.25 ± 20.99	209.24 ± 14.64	232.39 ± 2.8	202.89 ± 8.56	421.55 ± 15.1	336.87 ± 5.68	231.86 ± 1.76	206.36 ± 2.06	183.2 ± 6.23
122	unidentified	Unknown 21	58.74 ± 9.33	29.94 ± 2.45	27.77 ± 4.12	37.41 ± 1.22	23.68 ± 3.11	55.84 ± 3.69	22.08 ± 1.40	47.25 ± 3.25	15.41 ± 1.19	26.76 ± 0.37
123	unidentified	Unknown 22	26.95 ± 5.21	83.24 ± 3.63	55.57 ± 4.46	53.18 ± 3.41	87.90 ± 18.6	96.45 ± 3.78	106.34 ± 2.14	106.38 ± 4.91	94.16 ± 6.20	69.76 ± 5.76
124	925-78-0	3-Nonanone	17.11 ± 1.71	19.68 ± 1.20	33.30 ± 3.41	35.09 ± 2.02	26.88 ± 3.05	28.94 ± 2.76	26.76 ± 1.05	21.76 ± 3.48	18.98 ± 3.60	21.14 ± 3.70
125	102-13-6	2-Methylpropyl benzeneacetate	218.68 ± 76.15	130.11 ± 8.07	160.55 ± 8.12	120.13 ± 5.47	127.78 ± 11.1	139.02 ± 6.17	154.14 ± 9.84	143.58 ± 5.8	148.78 ± 7.26	163.4 ± 13.44
126	626-77-7	Hexanoic acid propyl ester	56.51 ± 18.35	119.32 ± 5.36	88.04 ± 1.19	101.97 ± 3.61	97.19 ± 7.34	85.58 ± 2.94	81.58 ± 3.48	120.5 ± 14.51	79.64 ± 12.74	81.35 ± 9.52
127	108-83-8	Isovalerone	19.75 ± 6.84	148.51 ± 44.31	276.85 ± 14.72	316.94 ± 13.6	373.29 ± 40.47	408.93 ± 12.6	405.6 ± 14.49	355.65 ± 20.11	385.04 ± 13.74	356.81 ± 28.07
128	123-51-3	3-Methyl-1-butanol	196.62 ± 9.23	166.14 ± 13.35	181.38 ± 3.47	190.05 ± 8.72	143.08 ± 8.95	133.63 ± 6.35	106.11 ± 6.21	131.06 ± 10.85	123.97 ± 7.95	99.16 ± 1.19
129	unidentified	Unknown 23	62.93 ± 6.54	257.71 ± 12.85	150.4 ± 4.76	263.92 ± 8.12	144.39 ± 8.04	85.77 ± 0.71	103.17 ± 3.55	77.01 ± 9.78	196.76 ± 8.95	47.39 ± 2.11
130	623-42-7	Methyl butanoate	536.37 ± 44.39	382.21 ± 20.97	312.93 ± 16.93	366.88 ± 15.86	296.88 ± 16.56	354.7 ± 8.23	241.17 ± 3.57	254.89 ± 7.2	298.8 ± 12.06	453.11 ± 31.21
131	96-17-3	2-Methyl butanal	165.95 ± 16.86	170.13 ± 15.37	178.74 ± 28.35	174.97 ± 7.56	51.77 ± 8.14	167.91 ± 6.72	49.33 ± 3.02	160.2 ± 10.84	63.42 ± 3.78	146.8 ± 5.22

A total of 132 volatile compounds were identified by GC-IMS, of which 109 could be characterised, including 26 esters, 17 aldehydes, 14 alcohols, 14 ketones, 9 olefins, 5 pyrazines, 4 ethers, 4 acids and 16 others.

**Figure 4:**
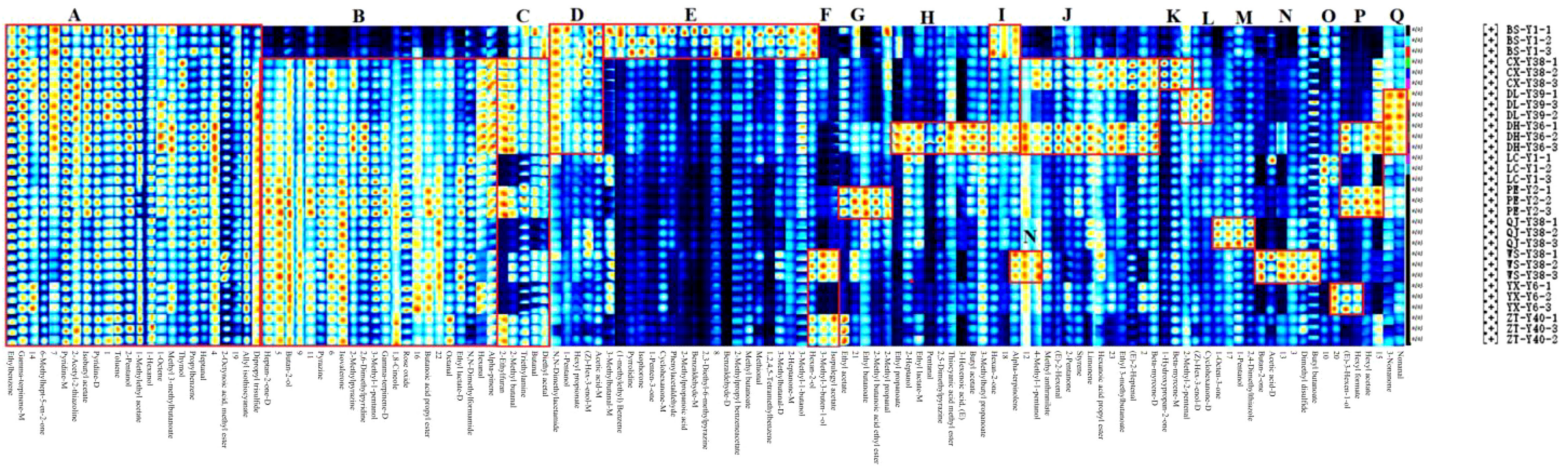
Fingerprinting of volatile organic compounds (VOCs) in cigar tobacco leaves from different regions.

In the fingerprinting results, each row and column represent all signal peaks of a sample or the same substance in different samples. The colour represents the content of the volatile compound; the lighter the colour, the higher the content. By comparing the colours in **Figure 4**, the substances with obvious differences in the fingerprints were classified into 17 zones from A to Q. The 24 compounds in zone A had small differences among all samples, and the differences between the compound contents of the BS-Y1 and the rest of the samples were obvious in zone B. 2-Methylpyrazine and 2,6-dimethylpyridine are considered the key aroma substances in tobacco (Yang et al., 2024). Compound contents of CX-Y38, DL-Y39, DH-Y36, PE-Y2, WS-Y38, and ZT-Y40 were higher in the C zone relative to other samples. The compound contents of the samples with high odour of N,N-dimethylbenzene were higher in zone C. The BS-Y1, CX-Y38, DL-Y39, and DH-Y36 contained high levels of the compounds in D zone, with the most pronounced differences in N,N-dimethylacetamide, which has a slightly ammonia odour. Zone E had compounds in BS-Y1 samples that were significantly higher than the contents of the other samples. Among the compounds identified, 3-methylbutanal-M, methyl butanoate, 3-methylbutanal-D and hexan-2-ol exhibited a fruity aroma. 2-Methylpropyl benzene acetate displayed a floral aroma, 2-heptanone-M exhibited a fruity and green aroma, and 3-methyl-1-butanol exhibited an apple brandy aroma. 2-Heptanone-M exhibited fruity and green characteristics, whereas 3-methyl-1-butanol displayed an apple brandy aroma, which may enhance the sensory appeal of the BS-Y1 samples relative to other samples. Zone F was the compounds in the hexan-2-ol and 3-methyl-3-ol compounds in the WS-Y38 and ZT-Y40 samples. Zone F was the compound in the hexan-2-ol and 3-methyl-methyl-methyl-methyl-3-ol samples and 3-methyl-3-buten-1-ol in the WS-Y38 and ZT-Y40 samples, all of which are fruity compounds. PE-Y2 samples had higher contents of ethyl acetate, ethyl butanoate and 2-methyl butanoic acid ethyl ester in zone G, all of which have floral and fruity aroma. The corresponding compounds in zone H were significantly more in the DH-Y36 sample than in the others. Zone I was the substance with higher content in the BS-Y1 and DH-Y36 samples, with hexan-2-one and α-terpinolene. Compounds in zone J are methyl anthranilate, (E)-2-hexenal, 2-pentanone, hexanoic acid propyl ester, ethyl 3-methylbutanoate, etc. The main aroma was fruity, and the content was higher in the CX-Y38 and DH-Y36 samples. Zones K to O corresponded to compound species that exhibit greater differentiation in the CX-Y38, DL-Y39, QJ-Y38, WS-Y38 and YX-Y6 samples compared to the other samples, respectively. The presence of dibutyl ketone, which exudes a creamy aroma, and butyl butanoate, which evokes a fruity aroma, in zone N may distinguish WS-Y38 from the other samples. 1-Octen-3 in zone M was characterised by the presence of compounds with low pleasantness, including one with an earthy and musty odour and 1-propanol with a musty and astringent odour. These contributed to a reduction in the overall aroma experience. Zone P was for substances with higher content in the DH-Y36 and PE-Y2 samples: (E)-3-hexen-1-ol with floral aroma, hexyl formate with fruity aroma and hexyl acetate with green and sweet aroma. Zone Q was for the substances with higher content in the DL-Y39 and DH-Y36 samples, and the substances with higher content in 3-nonanone, nonanone and nonaniline were the same as those in the DL-Y39 and DH-Y36 samples, respectively. Nonanone and nonanal have floral aroma.

**Figure 5:**
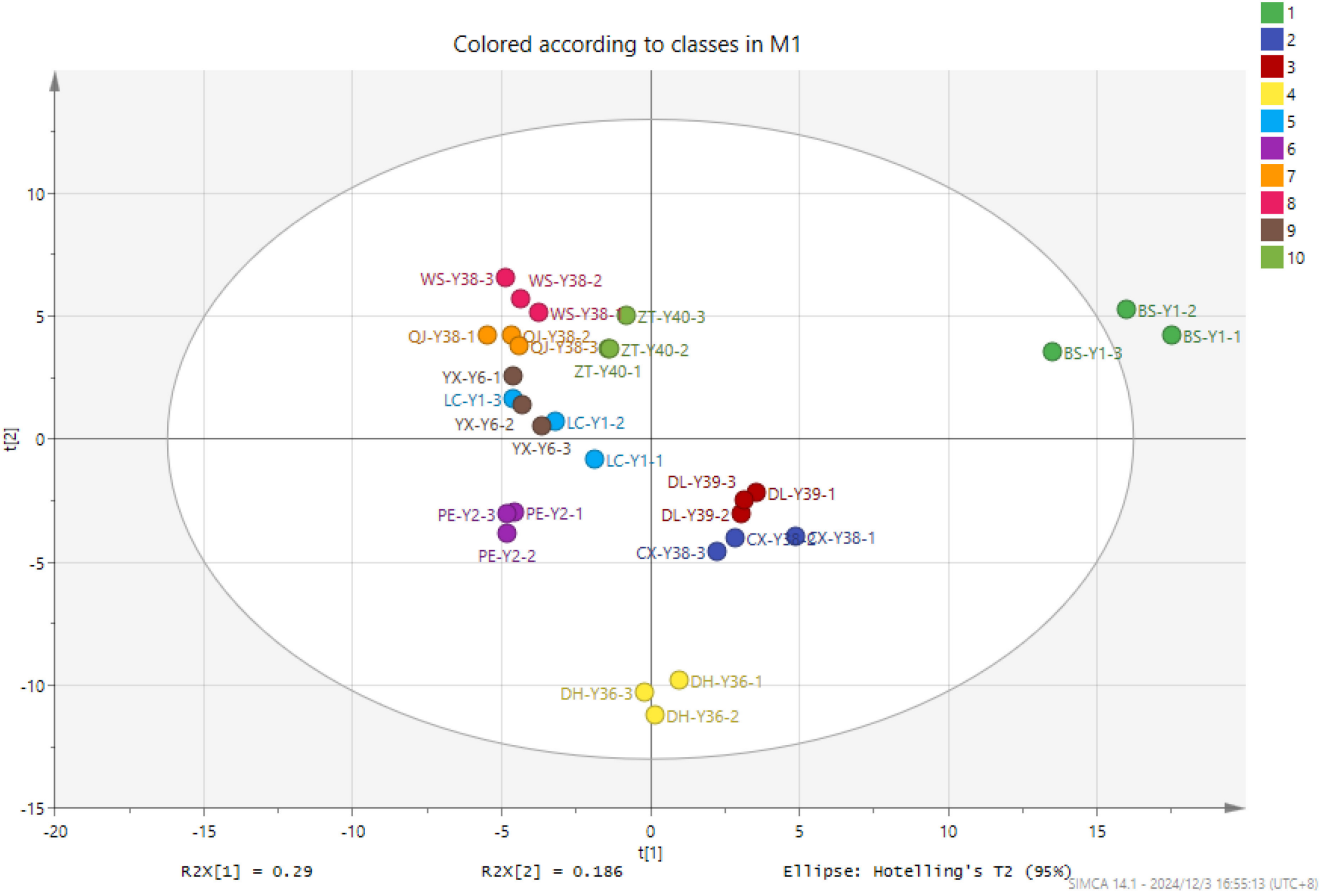
PCA of VOCs in cigar tobacco leaves of different origins.

PCA grouped 30 samples into 10 clusters, as illustrated in **Figure 5**. The cumulative contribution of the two principal components was 47%. The analysis of cigar tobacco samples from 10 distinct origins revealed a lack of clear differentiation in terms of the characteristic substances present, with a notable degree of aggregation between samples. However, significant differences were observed between the various sample groups. BS-Y1 was the most distant from all other samples, corresponding to the intuitive judgement in the fingerprint diagram. There were more differential compounds and differences in the content of compounds. The concentration of 20 compounds in BS-Y1 was markedly elevated compared to the remaining samples, whereas the levels of 20 compounds were notably diminished, exhibiting a considerable discrepancy compared to the other samples. The CX-Y38 and DL-Y39 cigar samples clustered into a single group, with the volatile compound types of the two samples exhibiting a high degree of proximity. The DL-Y39, LC-Y1, QJ-Y38, WS-Y38 and ZT-Y40 cigar samples were grouped together, indicating that the volatiles of the five samples were relatively similar. The DH-Y36 and PE-Y2 samples exhibited a distinct separation from the remaining samples, occupying a unique position within the data set. In the PCA1 dimension, the samples displayed a relatively minor divergence, except for BS-Y1.

### 3.2 Heatmap analysis of volatile compounds

**Figure 6:**
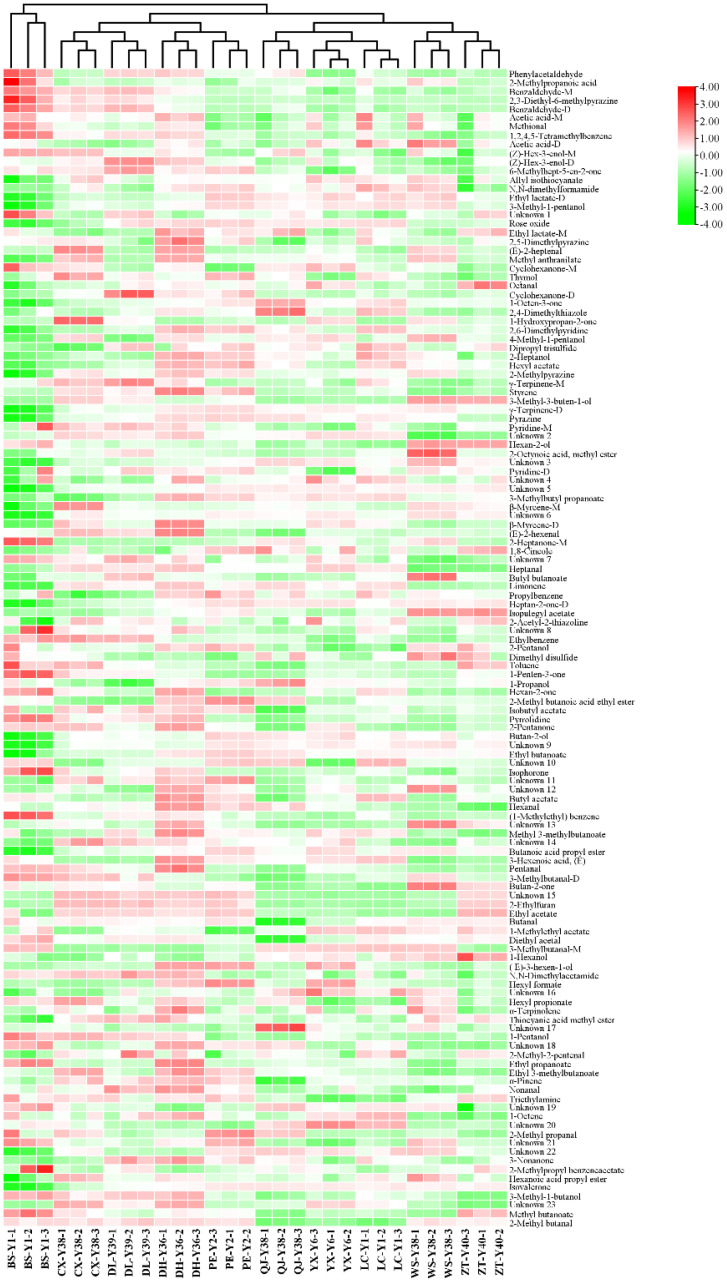
Heatmap of volatile compounds in cigar tobacco from different regions.

The volatile compounds of cigar tobacco leaves were analysed by heatmap clustering in 10 regions of Yunnan Province, namely Baoshan, Chuxiong, Dali, Dehong, Pu’er, Qujing, Yuxi, Lincang, Wenshan and Zhaotong. To better visualise the thermogram data, the volatile compound peak volumes were logarithmically processed and normalised as shown in **Figure 6**. Among the 10 origins, the Baoshan region was categorised separately, whereas the remaining 9 locations were categorised in another category. This was related to the high content of some compounds in cigar tobacco leaves in Baoshan, such as phenylacetaldehyde, 2-methylpropanoic acid, benzaldehyde-M, 2,3-diethyl-6-methylpyrazine, benzaldehyde-D, acetic acid-M, methional and 1,2,4,5-tetramethylbenzene. The remaining nine regions were grouped into two categories, with Chuxiong, Dali, Dehong and Pu’er in one category and Qujing, Yuxi, Lincang, Wenshan and Zhaotong in the other. Chuxiong, Dali, Dehong and Pu’er were at similar altitudes and were mostly hilly and mountainous, with unique regional microclimates that may influence the growth and metabolism of volatile compounds in cigar tobacco leaves. During the fermentation process, cigar tobacco leaves may undergo a Meladic reaction that produces various heterocyclic compounds and sulphur-containing compounds, which generally have a low aroma threshold and a significant impact on the aroma profile of cigar tobacco.

Results showed that cigar tobacco contained various pyrazines, pyridines and sulfides, including 2,3-diethyl-6-methylpyrazine, methional, allyl isothiocyanate, 2,5-dimethylpyrazine, 2,4-dimethylthiazole, 2,6-dimethylpyridine, dipropyl trisulfide, 2-methylpyrazine, pyrazine, pyridine-M, pyridine-D, 2-acetyl-2-thiazoline, dimethyl disulfide, pyrrolidine, 2-ethylfuran and thiocyanic acid methyl ester. Among them, the contents of 2,3-diethyl-6-methylpyrazine, methional and dimethyl disulfide in cigar tobacco in Baoshan were higher than those in other regions, whereas the contents of other substances were generally lower than those in other regions. In addition, ester compounds are the most diverse compounds produced in cigar tobacco leaves during the fermentation process, which are rich in aroma variations and strongly influence the aroma profile of cigar tobacco leaves.

Twenty-one ester compounds were detected in cigar tobacco, including ethyl lactate-D, ethyl lactate-M, methyl anthranilate, hexyl acetate, 2-octynoic acid, methyl ester, 3-methylbutyl propanoate, butyl butanoate, 2-methyl butanoic acid ethyl ester, ethyl butanoate, butyl acetate, methyl 3-methylbutanoate, butanoic acid propyl ester, ethyl acetate, 1-methylethyl acetate, hexyl formate, hexyl propionate, ethyl propanoate, ethyl 3-methylbutanoate, 2-methylpropyl acetate, benzene acetate, hexyl formate, ethyl propionate, ethyl 3-methylbutanoate, ethyl 3-methylbutanoate, methyl 3-methylbutanoate benzene acetate, hexanoic acid propyl ester and methyl butanoate. Similarly, the content of ester compounds in cigar tobacco leaves from Baoshan was generally lower than that of other regions, which is one of the reasons for the differences in the aroma quality of cigar tobacco leaves from different regions. Aromatic compounds, such as phenylacetaldehyde, benzaldehyde-M, 1,2,4,5-tetramethylbenzene, styrene and ethylbenzene, were generally higher in cigar tobacco leaves from Chuxiong, Dali, Dehong and Pu’er than from Qujing, Yuxi, Lincang, Wenshan and Zhaotong. The reasons for the differential distribution of volatile compounds in cigars in different regions may be related to cigar varieties, cultivation conditions, climatic conditions and geographical environment.

### 3.3 Metabolomics cluster analysis

According to GC-IMS and cluster analysis results, YX-Y6, PE-Y2 and DH-Y36 showed a great difference in organic volatiles, therefore, the metabolites of these 3 cigar tobacco samples were analysed emphatically. A total of 4993 metabolites were annotated in the three cigar tobacco leaves. To reflect the magnitude of the differences in metabolites among the three cigar tobacco leaves, orthogonal PLS-DA (OPLS-DA) of the metabolites of the three cigar tobacco leaves was performed in the positive- and negative-ion modes, and the results are shown in the figure above. PCA1 and PCA2 of OPLS-DA in the positive-ion mode explained 50.3% and 17.3% of the total variance, respectively, and PCA1 and PCA2 of OPLS-DA in the negative-ion mode explained 51.1% and 23.8% of the total variance, respectively. The R2X in OPLS-DA in the positive- and negative-ion modes were 0.939 and 0.859, R2 was 0.992 and 0.993 and Q2 was 0.928 and 0.964, respectively. It is generally believed that the model is more stable and reliable when R2 and Q2 are closer to 1. The above results indicated that the above model fits well and has a high explanatory rate and strong predictive ability and that there are substantial differences among the three types of cigar tobacco.

**Figure 7:**
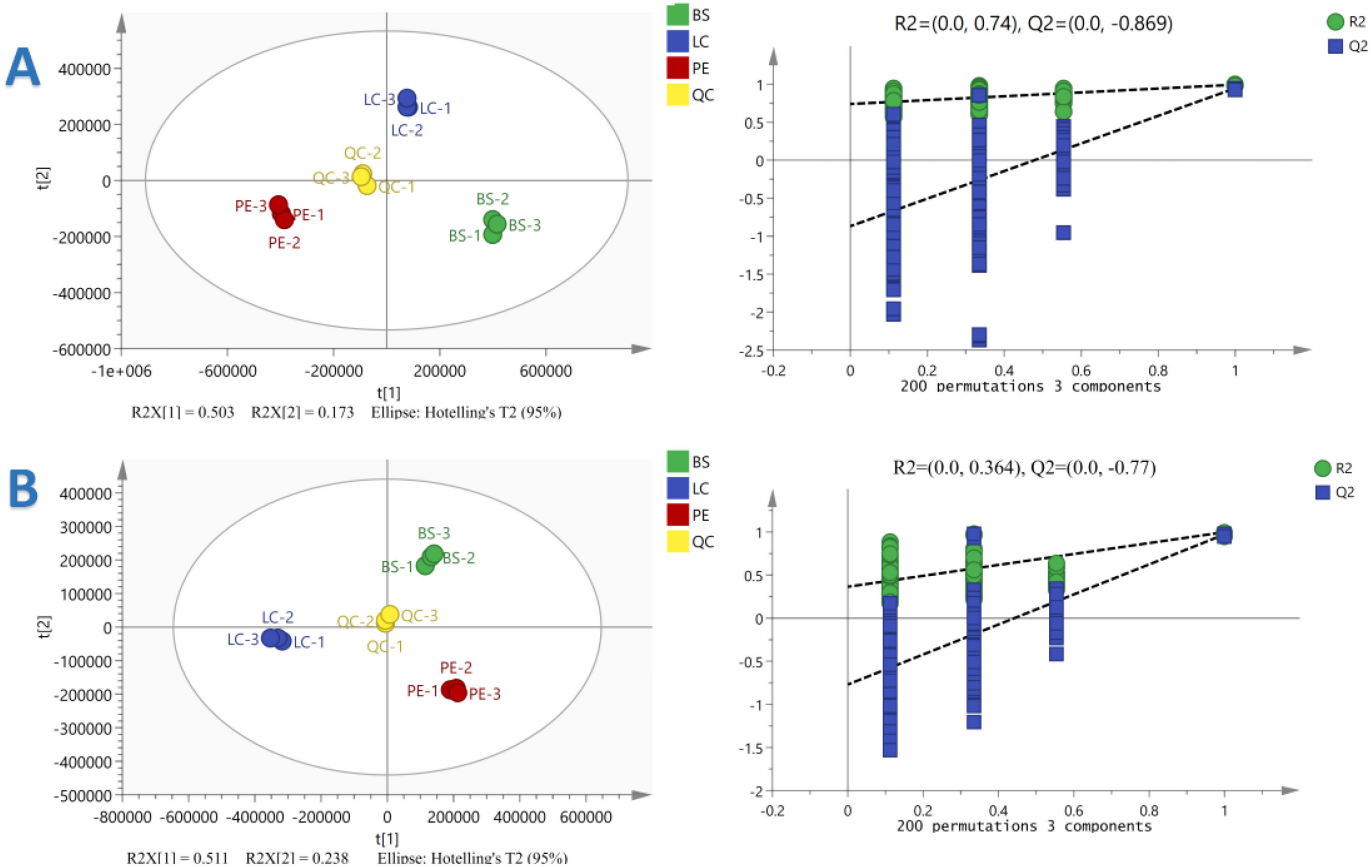
Orthogonal Partial Least Squares Discriminant Analysis (OPLS-DA) substitution test plots of three cigar tobacco leaves in the positive **(A)** and negative **(B)** ion modes.

To verify the ability of the established OPLS-DA model in terms of prediction and explanation, it was chosen to carry out the replacement test for verification. The OPLS-DA model data were subjected to 200 random response ordering tests, and the permutation test results were obtained based on the permutation retention as the horizontal coordinate and the R2 or Q2 values as the vertical coordinate, as shown in **Figure 7**. The R2 of the OPLS-DA model was 0.74 and 0.364 and the Q2 was −0.869 and −0.77 in the positive- and negative-ion modes, respectively, indicating that the OPLS-DA model showed an accurate fitting degree in the sample data, and there was no ‘overfitting’ phenomenon. This implied that the OPLS-DA model has strong explanatory and predictive ability and can effectively distinguish the three types of cigar tobacco. Therefore, the results can be used for subsequent differential metabolite analyses.

### 3.4 Analysis of differential metabolites

In OPLS-DA, the variable importance in projection (VIP) was calculated to measure the strength of the influence of the metabolite expression pattern on the classification discrimination of each group of samples, thus assisting in the screening of marker metabolites, and the metabolites with VIP>1 were usually used as differential metabolites or potential metabolites with VIP > 1 were usually used as differential metabolites or potential markers.

**Figure 8:**
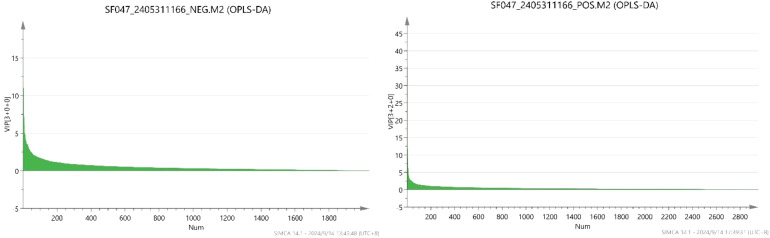
Metabolites VIP score.

The VIP scores of each metabolite are shown in **Figure 8**. By calculation, 2954 metabolites were detected in the positive-ion mode, among which 236 compounds with VIP>1 were detected. The 10 compounds with the largest VIP values were (S)-nicotine, L-pyroglutamic acid, L-phenylalanine, nornicotine, D-(+)-proline, indole-3-acetyl-L-aspartic acid, butyryl norfentanyl, carbendazim, pyraclostrobin and δ-valerolactam. Of the 2035 metabolites detected in the negative-ion mode, 245 compounds with VIP>1 were detected. The 10 compounds with the largest VIP values were 2-isopropylmalic acid, glyoxylic acid, 4-oxoproline, N-acetyl-DL-tryptophan, methoxyfenozide, 3-methylsalicylic acid, α-linolenic acid, N-acetyl-L-phenylalanine, azelaic acid and acetyl-N-formyl-5-methoxykynurenamine. A total of 470 metabolites had VIP>1 and could be used as differential metabolites in the positive- and negative-ion modes.

### 3.5 Metabolomics differential metabolite screening

**Figure 9:**
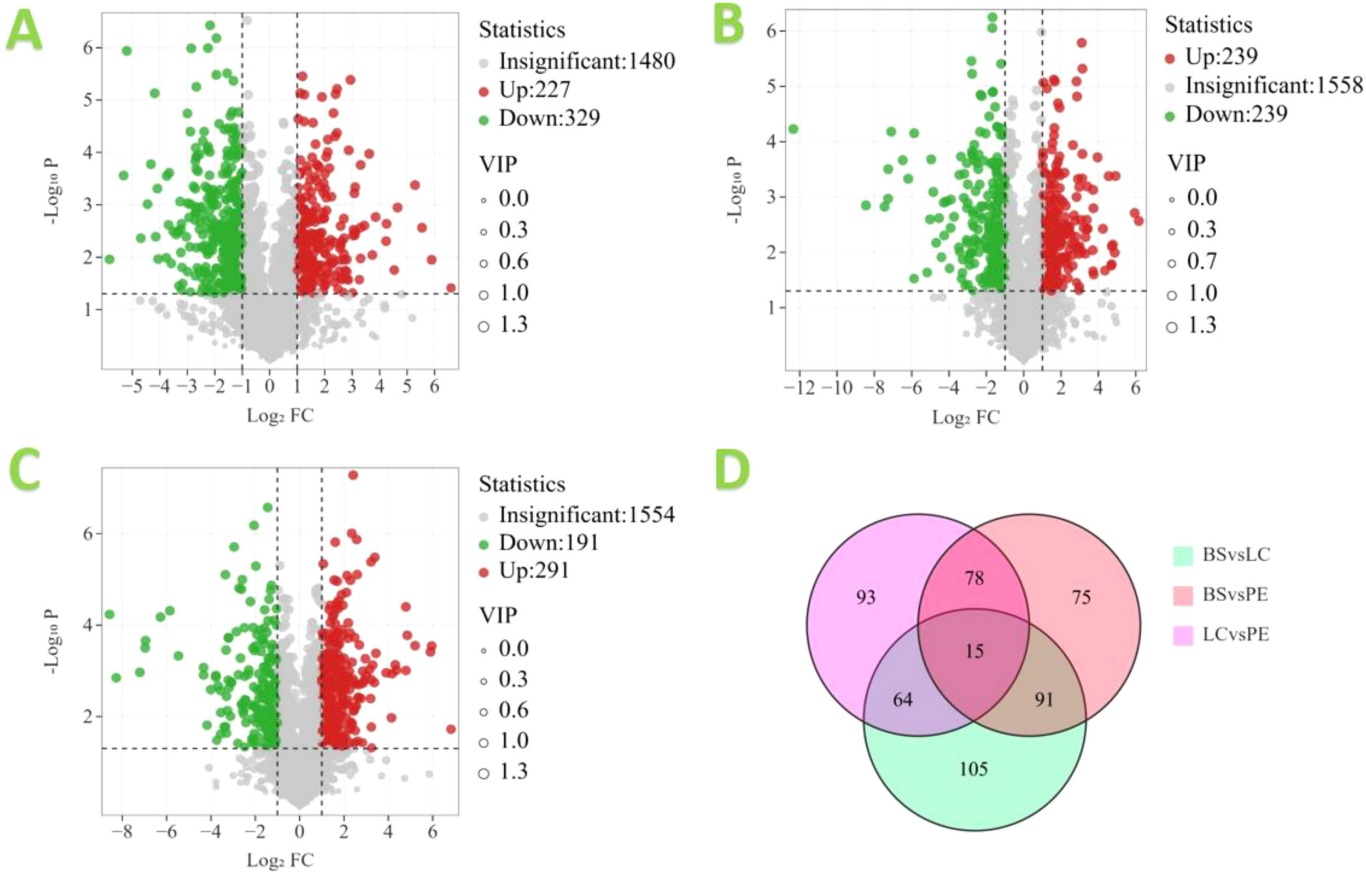
Volcano map of differential metabolites in different cigar tobacco leaves: **(A)** BS vs. LC, **(B)** BS vs. PE, **(C)** LC vs. PE and **(D)** Venn diagram.

Based on the OPLS-DA results, the three different cigar tobacco leaves were further screened for differential metabolites using FC, VIP and P-values from univariate analysis, and metabolites were considered differential metabolites when they simultaneously met FC > 1, VIP > 1 and P < 0.05 and volcano plots were drawn. The volcano diagram results are shown in **Figure 9**. In the BS vs. LC comparison, there were 556 differential metabolites, of which 227 metabolites were upregulated and 329 metabolites were downregulated; of which the upregulated metabolites indicated that 227 metabolites in BS were significantly higher than LC, and the downregulated metabolites indicated that 329 metabolites in BS were significantly lower than LC. In the BS vs. PE comparison, there were 478 differential metabolites, of which 239 metabolites were upregulated and 239 metabolites were downregulated. In the LC vs. PE comparison, there were 482 differential metabolites, of which 291 metabolites were upregulated and 191 metabolites were downregulated. Further analysis of the differential metabolites in the three comparison groups revealed that there were 15 common differential metabolites in all comparison groups, namely methoxyfenozide, dodecyl sulphate, myristyl sulphate, 2,3-dihydroxybenzoic acid, dinotefuran, 3-acetamidophenol, 1-(2-methylphenyl)hexahydropyrimidine-2,4,6-trione, 1-{[(2R,4S,5R)-5-{6-[4-(dimethylamino)phenyl]-2-methyl-4-pyrimidinyl}-1-azabicyclo[2.2.2]oct-2-yl]methyl}-3-isopropylurea, 3-chloro-N′-(pyridin-3-ylcarbonyl)benzohydrazide, T-2636F, (3-amino-3-carboxypropyl){[5-(6-amino-9H-purin-9-yl)-3,4-dihydroxytetrahydro-2-furanyl]methyl}methylsulfonium, cercosporene E, pterocidin, allahabadolactone A and (S)-(-)-scoulerine. Fifteen differential metabolites responded positively in all three tobacco species, suggesting they may be crucial metabolites in tobacco compounds.

### 3.6 Metabolic pathway analysis

A total of 433 metabolites were annotated for the Kyoto Encyclopedia of Genes and Genomes (KEGG) pathway in the BS-Y1 and LC-Y1 comparator groups, with 116 metabolic pathways. The three metabolic pathways with the highest P-values were alanine, aspartate and glutamate metabolism, nucleotide metabolism and degradation of flavonoids. The largest number of metabolites was observed in the biosynthesis of the secondary metabolites pathway, with 67 metabolites successfully annotated.

A total of 443 metabolites were annotated for the KEGG pathway in the BS-Y1 and PE-Y2 comparator groups, with 120 metabolites. The three metabolites with the highest P-values were alanine, aspartate and glutamate metabolism, nucleotide metabolism and degradation of flavonoids. The largest number of metabolites was in the biosynthesis of the secondary metabolites pathway, with 67 metabolites annotated. The most significant pathways were alanine, aspartate and glutamate metabolism, nucleotide metabolism and glyoxylate and dicarboxylate metabolism. The most abundant pathway was the biosynthesis of secondary metabolites, with 59 metabolites annotated.

A total of 423 metabolites were annotated for the KEGG pathway in the LC-Y1 and PE-Y2 samples, with 113 metabolism pathways. The three metabolism pathways with the highest P-values were alanine, aspartate and glutamate metabolism, glyoxylate and dicarboxylate metabolism and nucleotide metabolism. The largest number of metabolites was observed in the biosynthesis of the secondary metabolites pathway, with 61 metabolites annotated.

The pathways with high P-values in all three groups were alanine, aspartate and glutamate metabolism and nucleotide metabolism. The pathways with the most metabolites were all biosynthesis of secondary metabolites.

**Figure 10:**
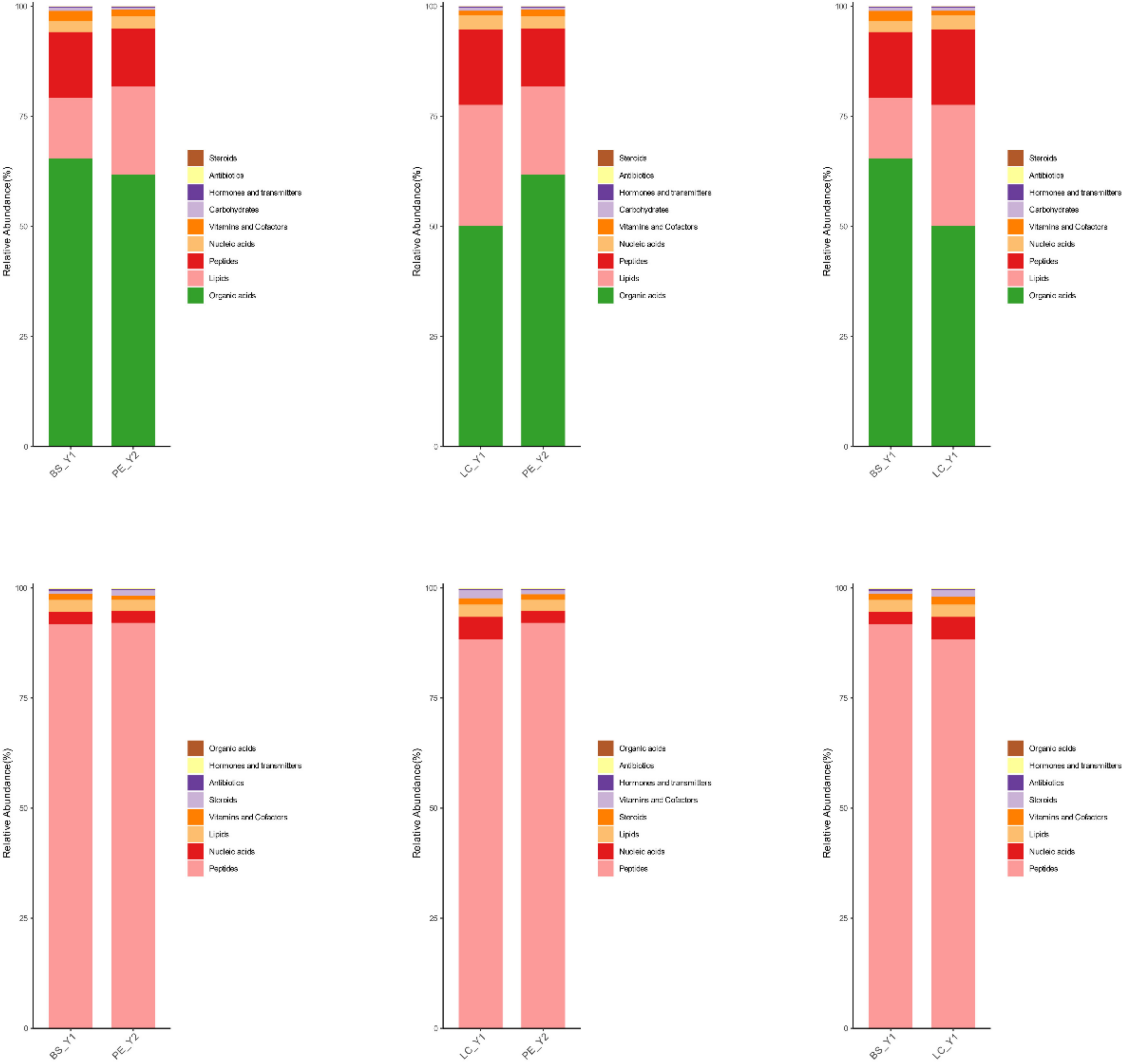
Metabolite stacking diagrams: top three for the negative-ion mode and bottom three for the positive-ion mode.

**Figure 10** shows the proportion of metabolite content. In the negative-ion mode, organic acids, lipids and peptides were the metabolites with the highest percentage. In the positive-ion mode, peptides and nucleic acids had the highest percentage. The two-way comparison results of the three groups of samples were similar. The metabolite species with high content in different cigar leaves exhibited a high degree of similarity. No significant differences were observed in the major metabolite species. The discrepancies in cigar tobacco between different origins may be attributed to the presence of lower metabolite levels or the existence of varying metabolite levels.

### 3.7 KEGG pathway enrichment analysis of differential metabolites

**Figure 11:**
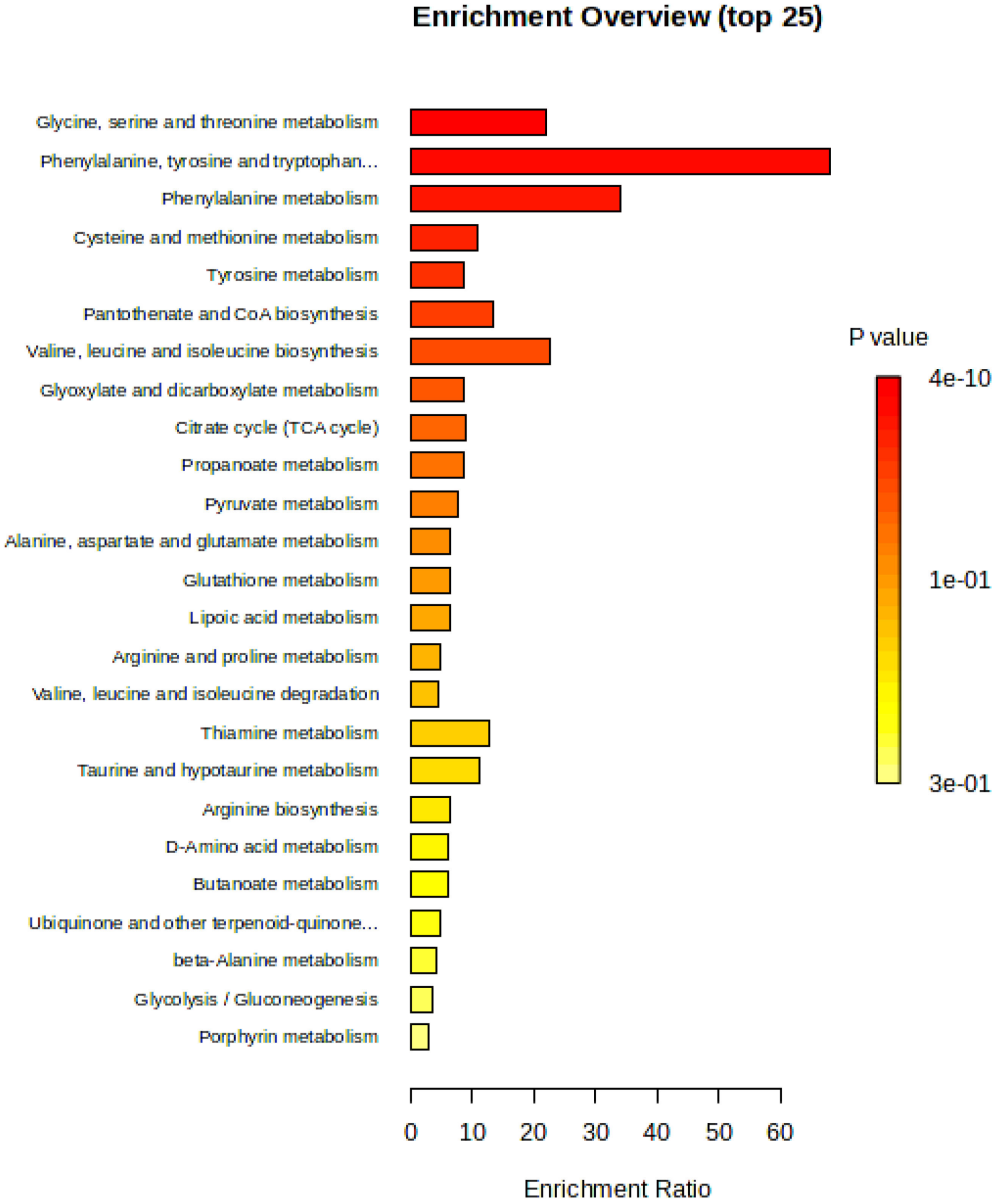
Overview of pathway enrichment analysis of differential metabolites in cigar tobacco leaves.

The KEGG pathway enrichment analysis of differential metabolites in three screened cigar tobacco leaves is shown. Regarding the annotation analysis and enrichment analysis pathways, only the number of metabolites or biomolecules involved in the pathway was considered, and the link of biomolecules involved in the pathway reaction was not considered. Typically, changes occurring in biomolecules at the centre of the reaction have a greater impact on the reaction than biomolecules in marginal or relatively isolated positions. The KEGG pathway topology is a method of evaluating the relative importance of metabolites or biomolecules to the pathway by means of weighted scores based primarily on the structure of the cyclic reaction of each in conjunction with the relative position of the biomolecules (Li et al., 2020). Using the pathway analysis function of MetaboAnalyst, pathway enrichment analysis was performed by KEGG IDs of differential metabolites. The pathways with top 25 significance were screened out, as shown in **Figure 11**, with the pathways increasing in P-value and decreasing in significance from top to bottom, from high to low, as follows: glycine, serine and threonine metabolism, phenylalanine, tyrosine and tryptophan biosynthesis, phenylalanine metabolism, cysteine and methionine metabolism, tyrosine metabolism, pantothenate and CoA biosynthesis, valine, leucine and isoleucine biosynthesis, glyoxylate and dicarboxylate metabolism, citrate cycle [tricarboxylic acid (TCA) cycle], propanoate metabolism, pyruvate metabolism, alanine, aspartate and glutamate metabolism, glutathione metabolism, lipoic acid metabolism, arginine and proline metabolism, valine, leucine and isoleucine degradation, thiamine metabolism, taurine and hypotaurine metabolism, arginine biosynthesis, D-amino acid metabolism, butanoate metabolism, ubiquinone and other terpenoid-quinone metabolism, β-alanine metabolism, glycolysis/gluconeogenesis and porphyrin metabolism.

The main enrichment pathway for differential metabolites in cigar tobacco samples is amino acid metabolism, including glycine, serine and threonine metabolism, phenylalanine metabolism, cysteine and methionine metabolism, tyrosine metabolism, alanine, aspartate and glutamate metabolism and arginine and proline metabolism. The biosynthesis of other amino acids includes phenylalanine, tyrosine and tryptophan biosynthesis, valine, leucine and isoleucine biosynthesis and arginine biosynthesis. The metabolism of cofactors and vitamins includes pantothenate and CoA biosynthesis. Carbohydrate metabolism includes glyoxylate and dicarboxylate metabolism, citrate cycle (TCA cycle), propanoate metabolism, pyruvate metabolism, lipoic acid metabolism, thiamine metabolism, taurine and hypotaurine metabolism, butanoate metabolism, ubiquinone and other terpenoid-quinone metabolism, glycolysis/gluconeogenesis and porphyrin metabolism. The metabolism of other amino acids includes glutathione metabolism, D-amino acid metabolism and β-alanine metabolism. Degradation of other amino acids includes valine, leucine and isoleucine degradation.

**Figure 12:**
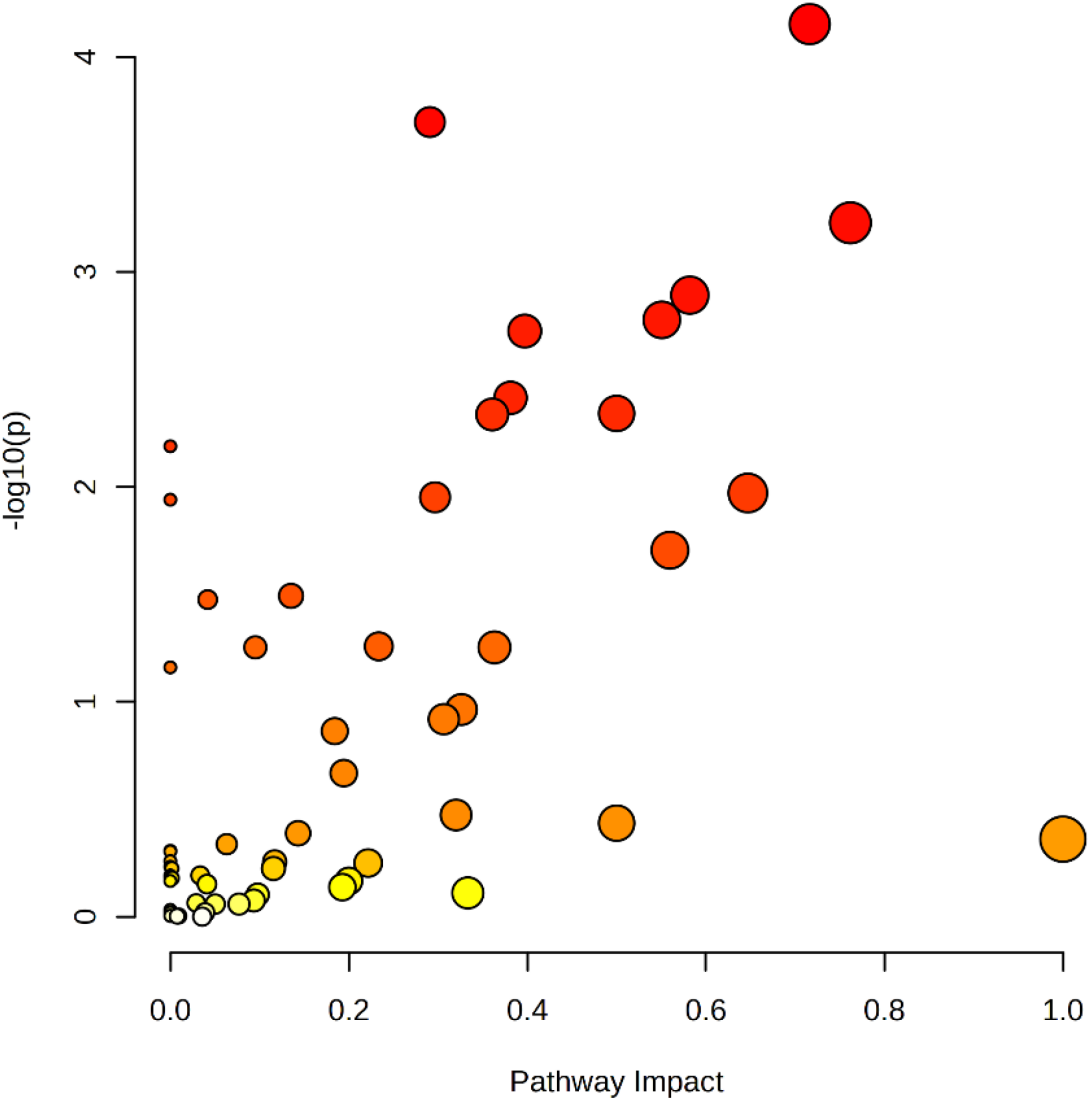
Bubble diagram of KEGG metabolic pathway in cigar tobacco leaves.

The relative magnitude of the effect of differential metabolites on the pathway was demonstrated by KEGG topology bubble plots, and the results are shown in **Figure 12** (Gong et al., n.d). Screening was performed based on impact > 0.5, and nine metabolic pathways were finally recognised as significantly different, namely linoleic acid metabolism, phenylalanine metabolism, alanine, aspartate and glutamate metabolism, vitamin B6 metabolism, glyoxylate and dicarboxylate metabolism, β-alanine metabolism, glycine, serine and threonine metabolism, phenylalanine, tyrosine and tryptophan biosynthesis and riboflavin metabolism.

In **Figure 12**, each bubble represents a KEGG pathway. The horizontal axis indicates the size of the impact value of the metabolite in the pathway, whereas the vertical axis indicates the significance of the metabolite’s enrichment in the pathway −log_10_ (P-value). The nodes are coloured from yellow to red, with redder nodes indicating smaller P-values and larger node radii indicating larger impact values. Larger dots farther away from the axes and darker in colour, indicating that the metabolic pathway is more affected. Amino acids are the key substances in the primary metabolism of tobacco and play an important role in the physiological activities of the plant (Oliva et al., 2021). Studies showed that the metabolite content of several amino acids associated with protein synthesis (phenylalanine, serine, glutamine, arginine, etc.) decreases gradually as the tobacco leaf matures (Jia et al., 2023). Serine was significantly negatively correlated with nicotine content. In tobacco metabolism, glycine, serine and threonine metabolic pathways may be related to the synthesis and catabolic process of nicotine, and these metabolic pathways can be used as a potential regulatory pathway of nicotine metabolism (Wang et al., 2016), consistent with this study. In the future, by observing experimental data, it is expected that the regulation of these different metabolic pathways will be used to discern the differences and the essential causes of the varieties of cigar tobacco and provide experimental ideas for the regulation of its flavour.

### 3.8 Metabolite classification analysis

**Figure 13:**
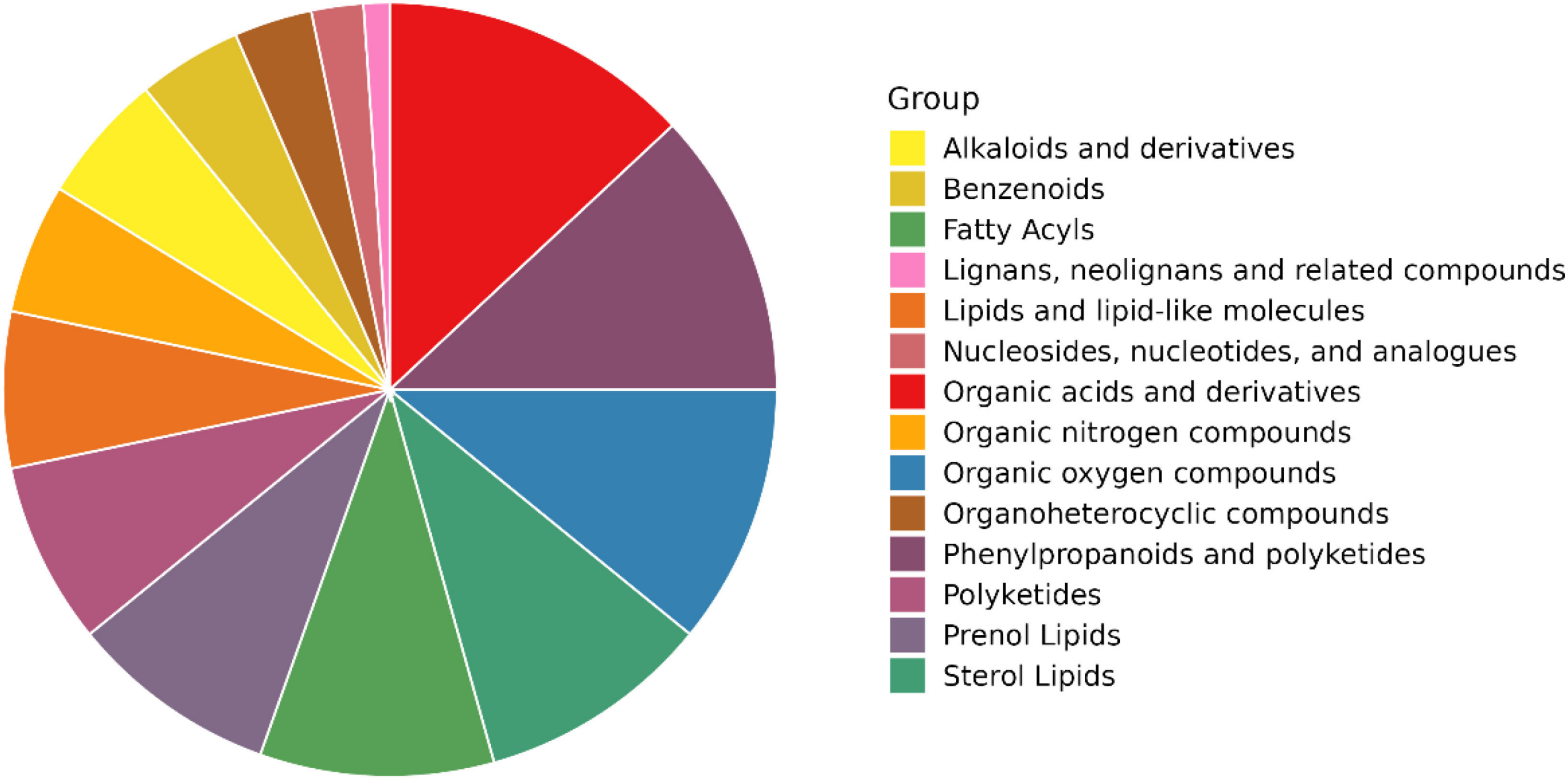
Pie chart of chemical classification of cigar tobacco metabolites.

All metabolites identified during the experiments (metabolites identified by combining positive and negative ions) were categorised and counted according to Chemical Taxonomy attribution information. The proportions of the number of each type of metabolite are shown in **Figure 13**. The following are alkaloids and derivatives, benzenoids, fatty acyls, lignans, neolignans and related compounds, lipids and lipid-like molecules, nucleosides, nucleotides and analogues, organic acids and derivatives, organic nitrogen compounds, organic oxygen compounds, organoheterocyclic compounds, phenylpropanoids and polyketides, polyketides, prenol lipids and sterol lipids.

Overall, the organic acids and their derivatives account for the highest percentage. Organic acids are a class of significant compounds widely present in tobacco, accounting for 12%–16% of the total dry matter of tobacco leaves (Lu et al., 2024). The content of differential metabolites of various acids in this study had the highest percentage, indicating that organic acids were fully transformed during the fermentation process of cigar tobacco leaves. In addition, the proportion of phenylacetones and polyketones was also high, which are precursors of tobacco aroma, and the degradation products are related to the aroma and aroma amount of tobacco, which is conducive to the enhancement of the quality and aroma, and increase the aroma amount. Alkaloids also occupy an important position in tobacco, and these substances have an impact on the quality of tobacco (Zhang et al., 2015). The main alkaloid in tobacco is nicotine, accounting for 90%–95% of the total alkaloids in tobacco. If the alkaloid content of cigar tobacco is high, it is drought-tolerant and disease-resistant; to a certain extent, it can inhibit pests and pathogens (Bian, 2021; Liu et al., 2024b). The presence of all these substances plays a role in enhancing the flavour and quality of cigar tobacco and has also been an important object of study during the tobacco research.

## 4 Conclusions

In this study, we conducted a comprehensive analysis of the volatile compound composition and metabolites in cigar tobacco leaves from different regions of Yunnan using GC-IMS and LC-MS metabolomics techniques. Our findings reveal significant regional variations in the chemical profiles of cigar tobacco leaves, which contribute to their distinct flavour characteristics. We identified 109 volatile compounds across the samples, including esters, aldehydes, alcohols, ketones, olefins, pyrazines, ethers, and acids. While certain compounds such as 1-methylethyl acetate, diethyl acetal, butanal, 1-hexanol, pyridine, and toluene were consistently present in high concentrations across all regions, we observed region-specific characteristics that clearly differentiated the samples. For instance, BS-Y1-1 exhibited high levels of 2,3-diethyl-6-methylpyrazine and phenylacetaldehyde, PE-Y2 contained the highest concentration of 3-methyl-1-pentanol, and WS-Y38 was distinguished by significantly elevated levels of butan-2-one. The KEGG pathway enrichment analysis indicated that amino acid metabolism, nucleotide metabolism, and glyoxylate and dicarboxylate metabolism were the predominant metabolic pathways influencing the flavour profile of Yunnan cigar tobacco leaves. Notably, tobacco leaves from the Baoshan region demonstrated distinct metabolic characteristics compared to those from other regions. These findings provide valuable insights into the chemical basis of regional differences in cigar tobacco leaves from Yunnan and contribute to our understanding of the factors that influence their quality and flavour profiles. The identified biomarkers and metabolic pathways could serve as important reference points for quality control, product authentication, and the development of region-specific tobacco products. Future research should focus on exploring the relationships between these chemical characteristics and sensory attributes, as well as investigating the environmental and agricultural factors that drive these regional differences.

## Data Availability

The original contributions presented in the study are included in the article/supplementary material. Further inquiries can be directed to the corresponding authors.

## References

[B1] Bian S. (2021). Mechanism of Tobacco Transcription Factor NtMYB305a inRegulating Nicotine Biosynthesis (Beijing, China: Chinese Academy of Agricultural Sciences).

[B2] Gong C. Xue D. He Z. Liu X. Xu D. Wu H.. (2024). Analysis of the effects of substances from cheyletusmalaccensis in grain based on untargeted metabolomics. J. Chin. Cereals Oils 40 (01), 14–22. doi: 10.20048/j.cnki.issn.1003-0174.000970

[B3] Han Y. Jiang S. Zhang E. Liu Z. Xue N. Li L. (2024). Analysis of volatile flavor characteristics of different types of air-Dried jerk based on electronic nose combined with HS-GC-IMS technology. Sci. Technol. Food Industry. 46, 1–11. doi: 10.13386/j.issn1002-0306.2024070090

[B4] Hou B. Xu J. Wang H. Hu Y. Wang Y. Qiu J. (2024). Analysis of quality differences of cigar tobacco from different producing areas. Tianjin Agric. Sci. 30, 74–78. doi: 10.3969/j.issn.1006-6500.2024.05.012

[B5] Hu J. Yu B. Ji L. Zhu B. Xue F. Fan W. (2023). Analysis and study on volatile aroma components of two types of cigars based on SHS-GC-MS and GC-IMS. Acta Tabacaria Sin. 29, 111–121. doi: 10.16472/j.Chinatobacco.2021.T0119

[B6] Jia Z. Zheng Q. Dai H. Zhang Y. Zhou H. Zhang S.. (2023). Differential analysis of metabolome of upper flue-cured tobacco leaf during ripening process. Tobacco Sci. Technol. 56, 1–10. doi: 10.16135/j.issn1002-0861.2022.0692

[B7] Joshi R. Sharma P. Sharma V. Prasad R. Sud R. Gulati A. (2013). Analysis of the essential oil of large cardamom (Amomum subulatum Roxb.) growing in different agro-climatic zones of Himachal Pradesh, India. J. OF THE Sci. OF Food AND Agric. 93, 1303–1309. doi: 10.1002/jsfa.5886 23023817

[B8] Li X. Bin J. Yan X. Ding M. Yang M. (2022). Application of chromatographic technology to determine aromatic substances in tobacco during natural fermentation: A review. Separations 9, 187. doi: 10.3390/separations9080187

[B9] Li X. Chen A. Pang S. Haiyan Z. Dongmei Q. Jun Q.. (2024b). Comparative study on metabolic characteristics of flavonoids in tobacco leaves from different growing areas in Sichuan Province. Tobacco Sci. Technol. 57, 33–42. doi: 10.16135/j.issn1002-0861.2023.0506

[B10] Li J. Hu Y. Lu M. Du X. Chen Y. Hu B.. (2024a). Metabolomic analysis of metabolic differences and formation mechanisms in open fire-cured tobacco leaves. J. Yunnan Agric. University(Natural Science) 39, 80–90. doi: 10.12101/j.issn.1004-390X(n).202305026

[B11] Li S. Li J. Gao X. Liu W. Chang S. Ou Z.. (2023). Analysis of characteristic “Glutinous rice”-like aroma-active odorants in flue-cured tobacco leaves based on HS-GC-IMS combined with ROAV. Chin. Tobacco Sci. 44, 75–83. doi: 10.13496/j.issn.1007-5119.2023.06.011

[B12] Li R. Zhang Y. Wang Y. Huang K. Yang Q. Zhang T.. (2020). Aqueous extract of Fritillariae cirrhosae induces cellular apoptosis through activation of STATs-mediated immunomodulation. J. Ethnopharmacology 261, 112338. doi: 10.1016/j.jep.2019.112338 31669666

[B13] Liu Y. Fang D. Xu H. Tong Z. (2024b). QTL mapping of alkaloids in tobacco. Acta Agronomica Sin. 50, 42–54. doi: 10.3724/SP.J.1006.2024.34047

[B14] Liu F. Wu Z. Zhang X. Xi G. Zhao Z. Lai M.. (2021). Microbial community and metabolic function analysis of cigar tobacco leaves during fermentation. MicrobiologyOpen 10, e1171. doi: 10.1002/mbo3.1171 33970539 PMC8483401

[B15] Liu X. Zhang L. Sun H. Zhang Z. He P. Pengfei Y.. (2024a). Discrimination of different degrees of mildewed tobacco based on HS-GC-IMS technique. J. Instrumental Anal. 43, 1433–1441. doi: 10.12452/j.fxcsxb.24042901

[B16] Lu A. Zhang L. Wang P. Chen X. Du S. Li X.. (2024). Differences in leaf metabolites of three cigar varieties before and after leaf fermentation. J. Northwest A F University(Natural Sci. Edition) 52, 1–12. doi: 10.13207/j.cnki.jnwafu.2024.12.006

[B17] Maurya A. Aggarwal G. Vashisath S. Kumar V. Agnihotri V. (2023). Chemodiversity and α-glucosidase activity of eucalyptus species from northwestern himalaya, India. Chem. BIODIVERSITY 20. doi: 10.1002/cbdv.202300223 37463873

[B18] Maurya A. K. Devi R. Kumar A. Koundal R. Thakur S. Sharma A.. (2018). Chemical composition, cytotoxic and antibacterial activities of essential oils of cultivated clones of *juniperus communis* and wild *juniperus* species. Chem. Biodiversity 15, e1800183. doi: 10.1002/cbdv.201800183 29956891

[B19] Maurya A. Vashisath S. Aggarwal G. Yadav V. Agnihotri V. (2022). Chemical diversity and a-glucosidase inhibitory activity in needles essential oils of four pinus species from Northwestern Himalaya, India. Chem. BIODIVERSITY 19. doi: 10.1002/jsfa.5886 36395372

[B20] Mukherjee A. G. Gopalakrishnan A. V. Jayaraj R. Ganesan R. Renu K. Vellingiri B.. (2023). Recent advances in understanding brain cancer metabolomics: a review. Med. Oncol. 40. doi: 10.1007/s12032-023-02109-3 37402029

[B21] Oliva M. Guy A. Galili G. Dor E. Schweitzer R. Amir R.. (2021). Enhanced production of aromatic amino acids in tobacco plants leads to increased phenylpropanoid metabolites and tolerance to stresses. Front. Plant Sci. 11. doi: 10.3389/fpls.2020.604349 PMC7835393 33510749

[B22] Shen S. Zhan C. Yang C. Alisdair R F. Luo J. (2023). Metabolomics-centered mining of plant metabolic diversity and function: Past decade and future perspectives. Mol. Plant 16, 43–63. doi: 10.1016/j.molp.2022.09.007 36114669

[B23] Wang S. Chen H. Sun B. (2020). Recent progress in food flavor analysis using gas chromatography–ion mobility spectrometry (GC–IMS). Food Chem. 315. doi: 10.1016/j.foodchem.2019.126158 32014672

[B24] Wang W. Xi F. Yang S. Jiang J. Wang F. (2016). Progress on nicotine metabolism regulation in tobacco. Subtropical Agric. Res. 12, 62–67. doi: 10.13321/j.cnki.subtrop.agric.res.2016.01.010

[B25] Wang Z. Zhang M. Liu W. Shi H. Wei J. Zhao Y. (2024). Study on effect of light duration on growth of flue-cured tobacco based on metabolomics. Jiangsu Agric. Sci. 52, 94–100. doi: 10.15889/j.issn.1002-1302.2024.14.013

[B26] Wu W. Tang X.-P. Chao Y. Liu H.-B. (2013). Investigation of ecological factors controlling quality of flue-cured tobacco (Nicotiana tabacum L.) using classification methods. Ecol. Informatics. 16, 53–61. doi: 10.1016/j.ecoinf.2013.04.008

[B27] Xiaohong R. Gang C. Haiyan M Tianhe P. Yanfang L. Jianjun Y (2010). Research on the effect of cell wall matter components on the tobacco quality. Chin. Agric. Sci. Bull. 26, 113–116.

[B28] Yang L. Liu L. Ji L. Jiang C. Jiang Z. Li D.. (2024). Analysis of differences in aroma and sensory characteristics of the mainstream smoke of six cigars. Heliyon 10, e26630. doi: 10.1016/j.heliyon.2024.e26630 38434019 PMC10906419

[B29] Yang X. Zhang T. Yang D. Xie J. (2023). Application of gas chromatography-ion mobility spectrometry in the analysis of food volatile components. Acta Chromatographica 35, 35–45. doi: 10.1556/1326.2022.01005

[B30] Yin J. (2021). Application and development trends of gas chromatography–ion mobility spectrometry for traditional Chinese medicine, clinical, food and environmental analysis. Microchemical J. 168. doi: 10.1016/j.microc.2021.106527

[B31] Zhang l Ji H. Huang X. Wang F. Liu J. (2015). Plant metabolomics and its application in tobacco. Acta Tabacaria Sin. 21, 126–134. doi: 10.16472/j.Chinatobacco.2014.430

[B32] Zhang T. Zhang A. Qiu S. Yang S. Wang X. (2016). Current trends and innovations in bioanalytical techniques of metabolomics. Crit. Rev. Analytical Chem. 46, 342–351. doi: 10.1080/10408347.2015.1079475 26337255

[B33] Zhao M. Wang B. Li F. Qiu L. Li F. Wang S.. (2007). Analysis of bacterial communities on aging flue-cured tobacco leaves by 16S rDNA PCR–DGGE technology. Appl. Microbiol. Biotechnol. 73, 1435–1440. doi: 10.1007/s00253-006-0625-x 17043820

